# FKBP9 promotes the malignant behavior of glioblastoma cells and confers resistance to endoplasmic reticulum stress inducers

**DOI:** 10.1186/s13046-020-1541-0

**Published:** 2020-02-28

**Authors:** Huizhe Xu, Peng Liu, Yumei Yan, Kun Fang, Dapeng Liang, Xiukun Hou, Xiaohong Zhang, Songyan Wu, Jianmei Ma, Ruoyu Wang, Tao Li, Haozhe Piao, Songshu Meng

**Affiliations:** 1 Institute of Cancer Stem Cell, Dalian Medical University Cancer Center, 9 Lvshun Road South, Dalian, 116044 Liaoning Province China; 2 Department of General Surgery, Shenzhen University General Hospital, No. 1098, Xueyuan avenue, Shenzhen, 518055 China; 3 The First Department of Ultrasound, The First Affiliated Hospital, Dalian Medical University, No. 222 Zhongshan Road, Dalian, 116021 Liaoning Province China; 4 Department of Anatomy, Dalian Medical University, 9 Lvshun Road South, Dalian, 116044 Liaoning Province China; 5 Department of Oncology, Affiliated Zhongshan Hospital of Dalian University, No.6 Jiefang Street, Dalian, 116001 Liaoning Province China; 6 Department of Neurosurgery, The First Affiliated Hospital of Dalian Medical University, No. 222 Zhongshan Road, Dalian, 116011 Liaoning Province China; 7 Department of Neurosurgery, Cancer Hospital of China Medical University, Liaoning Cancer Hospital & Institute, No. 44 Xiaoheyan Road, Dadong District, Shenyang, 110042 Liaoning Province China

**Keywords:** Endoplasmic reticulum (ER) stress, FK506-binding protein 9, Unfolded protein response (UPR), Glioma, IRE1α-XBP1

## Abstract

**Background:**

FK506-binding protein 9 (FKBP9) is amplified in high-grade gliomas (HGGs). However, the roles and mechanism(s) of FKBP9 in glioma are unknown.

**Methods:**

The expression of FKBP9 in clinical glioma tissues was detected by immunohistochemistry (IHC). The correlation between FKBP9 expression levels and the clinical prognosis of glioma patients was examined by bioinformatic analysis. Glioblastoma (GBM) cell lines stably depleted of FKBP9 were established using lentiviruses expressing shRNAs against FKBP9. The effects of FKBP9 on GBM cells were determined by cell-based analyses, including anchorage-independent growth, spheroid formation, transwell invasion assay, confocal microscopy, immunoblot (IB) and coimmunoprecipitation assays. In vivo tumor growth was determined in both chick chorioallantoic membrane (CAM) and mouse xenograft models.

**Results:**

High FKBP9 expression correlated with poor prognosis in glioma patients. Knockdown of FKBP9 markedly suppressed the malignant phenotype of GBM cells in vitro and inhibited tumor growth in vivo. Mechanistically, FKBP9 expression induced the activation of p38MAPK signaling via ASK1. Furthermore, ASK1-p38 signaling contributed to the FKBP9-mediated effects on GBM cell clonogenic growth. In addition, depletion of FKBP9 activated the IRE1α-XBP1 pathway, which played a role in the FKBP9-mediated oncogenic effects. Importantly, FKBP9 expression conferred GBM cell resistance to endoplasmic reticulum (ER) stress inducers that caused FKBP9 ubiquitination and degradation.

**Conclusions:**

Our findings suggest an oncogenic role for FKBP9 in GBM and reveal FKBP9 as a novel mediator in the IRE1α-XBP1 pathway.

## Background

FK506-binding protein 9 (FKBP9) belongs to a family of immunophilins that are able to bind to FK506, an immunosuppressive drug [1]. FKBPs are known to be involved in multiple biological processes, such as playing its immunosuppressive roles and inactivating the nuclear factor of activated T-cells [1, 2]. In addition, FKBPs have been implicated in cancer development. For instance, the expression of FKBP51 (also referred as FKBP5) is highly upregulated in glioma specimens and high FKBP51 expression is positively correlated with glioma grade [3]. Moreover, FKBP51 is highly expressed in prostate cancer, lymphoma, and melanoma and its expression correlates with metastatic potential in melanoma and prostate cancer [4–7]. In neuroblastoma and lung cancer, FKBP12 acts as an antagonist of the MDM2-p53-feedback loop during cellular stress and DNA damage [8]. Of note, several members of the FKBP family, including FKBP9 (also referred to as FKBP60 or FKBP63), FKBP13, FKBP23 and FKBP65, localize to the endoplasmic reticulum (ER) as they contain the ER retention motif H/R/KDEL. Among the ER-residing FKBPs, FKBP65 has been implicated in several types of cancer such as high-grade ovarian serous carcinoma [9], melanoma [10] and renal cell carcinoma [11]. Of interest, FKBP9 is highly amplified in gliomas across most cancer types, as revealed by an initial survey of the TCGA database [12]. Moreover, a mutation of methionine (M) 541 into isoleucine (I) in FKBP9 was found in clinical glioma tissue samples [13]. In addition, the FKBP9 mutation was also reported to be associated with disease-free survival rates in pheochromocytoma or paraganglioma [14]. However, the precise role and mechanism(s) of action of FKBP9 in glioma remain completely unknown.

The unfolded protein response (UPR), frequently activated upon ER stress, functions as an adaptive cellular program to sustain protein homeostasis and to protect cells from prolonged or severe ER stress–triggered cell death [15]. Nonetheless, if ER homeostasis cannot be achieved, then the UPR could lead the cell towards cell death. The UPR involves the activation of three ER-resident transmembrane protein sensors: inositol requiring enzyme 1α (IRE1), activating transcription factor 6 (ATF6), and protein kinase R–like ER kinase (PERK). A high level of basal UPR is frequently found in a wide range of primary human tumors, including glioblastoma (GBM, WHO grade IV gliomas) and carcinomas of the liver and stomach [16–19]. In the context of GBM, UPR signaling can mediate both pro-survival and pro-death mechanisms [20]. Of importance, UPR-modulating drugs, including ER stress-inducing compounds, have emerged as promising candidates to combine with TMZ, a stand-alone reagent, for the treatment of GBM [20].

In this study, we report that FKBP9 is upregulated in human GBM samples and correlates with poor prognosis. Moreover, by gain- and loss-of-function studies we demonstrate an oncogenic role of FKBP9 in GBM progression. In addition, we show that FKBP9 expression confers GBM cell resistance to ER stress inducer-triggered cell death by modulating IRE1 signaling. Thus, this study provides evidence that FKBP9 exhibits oncogenic effects on GBM progression and uncovers the role of FKBP9 in regulating UPR signaling.

## Materials and methods

### Cell lines, plasmids and transfection

Human embryonic kidney 293 T (HEK-293 T), rat glioma cell line C6, human GBM cell lines A-172, Hs 683, LN-18, LN-229, T98G, U-87 MG were obtained from the American Type Culture Collection (ATCC). SF-539 and SF-767 GBM cell lines were obtained from the cell bank of the Chinese Academy of Science. All cell lines were maintained at 37 °C in a humidified incubator with 5% CO_2_. HEK-293 T, C6, A-172, Hs 683, LN-18 and LN-229 were cultured with DMEM (Gibco). T98G and U-87 MG were cultured with EMEM (Gibco). All the GBM cell lines used in this study carry mRNA encoding the wild type FKBP9.

Full length of FKBP9 cDNA was obtained from the Center for Cancer Systems Biology (CCSB)-Broad Lentiviral Expression Library (#11328). V5-tagged FKBP9 wide type, deletion and point mutants were constructed by standard molecular cloning procedures. Adenovirus FKBP9 (Ad-FLAG-FKBP9) and control virus (Ad-vector) were purchased from Vigene Biosciences (VH808170). Infection of adenovirus was performed according to the manufacturer’s instructions. Lipofectamine 3000 (Invitrogen) was used for transfection.

### Antibodies and reagents

Anti-V5, FLAG and GAPDH antibodies were purchased from Proteintech. Anti-α-Tubulin and anti-HA antibodies were purchased from Sigma. Anti-Sox2, Nanog and Oct4 antibodies were purchased from Abcam. The following antibodies were used: anti-FKBP9 (Invitrogen), anti-Calnexin (Santa Cruz), anti-Nestin (R&D Systems), anti-pmTOR (Invitrogen), anti-pP70S6K (Millipore), anti-pERK1/2 (Promega), anti-p65 (EPITOMICS), anti-ASK1 (Santa Cruz), anti-pASK1 (Santa Cruz), anti-pIRE1α (NOVUS). Other antibodies for immunoblotting were purchased from Cell Signaling Technology. Aggresome Detection Kit was purchased from Abcam. Thapsigargin (Tg) and tunicamycin (Tm) were purchased from Apexbio. Proteasome inhibitor MG132 and lysosomal inhibitors Bafalomycin A1 (Baf A1) and chloroquine (CQ) were obtained from Sigma. Drugs were dissolved and stored at − 20 °C or − 80 °C according to the instructions.

### Bioinformatics analysis

RNA was extracted from shControl and shFKBP9 cells and RNA-Seq was performed by the Novogene Corporation (Beijing, China). The sequencing libraries were constructed using NEBNext® UltraTM RNA Library Prep Kit for Illumina® (NEB, USA) according to the manufacturer’s instructions. Clean data were obtained by removing reads containing adapter, reads containing ploy-N and low quality reads from raw fastq data using in-house perl scripts. Paired-end clean reads were aligned to the reference genome hg38 using Hisat2 v2.0.5. feature Counts v1.5.0-p3 was used to generate gene-level count matrix as input for edgeR’s statistical model. Differential expression analysis between shFKBP9 and shControl cells was performed using the edgeR package. The *P* values were adjusted using the Benjamini & Hochberg method. Corrected *P*-value of 0.05 and absolute fold change of 2 were set as the threshold for significantly differential expression. The association between FKBP9 and potential biological mechanisms was analyzed with GSEA v3.0 using the gene set Protein processing in endoplasmic reticulum(hsa04141) from the Molecular Signatures Database (MSigDB) as a reference. Metrics for ranking the key mRNAs were calculated based on fold change in FKBP9 knockdown vs control SF-539 cells.

To comprehensively explore the expression pattern and prognostic implications of FKBP9 in gliomas, preprocessed RNA-seq and corresponding clinical data were downloaded from UCSC XENA (TCGA-GBMLGG) (https://xenabrowser.net/datapages/) and CGGA (http://www.cgga.org.cn). Microarray data for Repository for Molecular Brain Neoplasia Data (Rembrandt) was obtained from Gene Expression Omnibus (GEO) data repository with accession number GSE108474. Raw data was processed using rma function from Bioconductor rma package with the default setting. The mas5calls function from affy package was used to generate present/marginal/absent calls for all sample replicates of all probesets. Each “present” call was assigned a value of 1.0, “marginal” was assigned a value of 0.5, and “absent” a value of 0. For averages > 0.4, the probeset was considered reliable detection. Non-specific probesets that ended with “_x_at” were excluded. Filtered probesets were then mapped to the corresponding genes using hgu133plus2.db annotation package. Multiple probesets mapped to the same gene were aggregated as average signal intensity value. Statistical analyses were performed using the R statistical software. Survival analysis was conducted via the ‘survminer’ package. Gliomas patients were categorized into High and Low FKBP9 expression group using the median expression as cutoff points and survival curves were based on Kaplan-Meier estimates. Differential FKBP9 expression in GBM and LGG was determined by Non-parametric Mann-Whitney test. Pearson’s correlation between mRNA expression of FKBP9 and stem marker was calculated.

### RNA interference

Two siRNA oligonucleotides of p38 were used:

5′- AUGAAUGAUGGACUGAAAUGGUCUG -3′;

5′- GGACCUCCUUAUAGACGAAUU − 3′.

Two siRNA oligonucleotides of ASK1 were used:

5′- CCGGGAAUCUAUACUCAAUTT -3′;

5′- GCAUUUGAAUCUGAGCCAATT − 3′. A scrambled siRNA: 5′-UUCUCCGAACGUGUCACGUTT-3′ was used as a negative control. The silencing efficiency was detected by immunoblotting assay.

### Lentiviral constructs and stable cell lines

The lentiviral constructs, pGIPZ-CTRL and FKBP9 shRNA were purchased from the Dharmacon™ GIPZ™ Lentiviral shRNA Library (#11328). Lentivitral expression plasmids (PCDH-FKBP9-WT, PCDH-FKBP9-M541I and PCDH-FKBP9-K265R) were produced by PCR. Lentiviral plasmids together with package plasmids (PsPAX_2_ and PMD_2_G) were transfected into HEK 293 T cells to produce viruses. Stable clones were selected using puromycin (5 μg/mL for LN-229, 2 μg/mL for SF-539, 2 μg/mL for T98G and 1 μg/mL for U-87 MG) to establish cell lines with stable knockdown FKBP9 or overexpression of V5-tagged FKBP9.

### Cell viability, colony formation, cell invasion and 3D culture assays

Cells were seeded (2000 cells/well) in 96-well plates for the indicated time, and cell viability was detected with CCK8 kit (MCE/Y-K0301) according to the manufacturer’s protocol. Colony formation, cell invasion and 3D culture assays were performed as previously described [21].

### Chick embryo chorioallantoic membrane (CAM) assays

Fertilized chicken eggs (purchased from MERIAL, Beijing) were incubated at 37 °C and 65% humidified atmosphere during 10 days. On day 10, cells were deposited on the surface of the CAM at an amount of 1X10^6^. Tumor growth and metastasis *in ovo* were determined at day 17.

### Confocal microscopy, Immunoprecipitation, Immunoblotting and immunohistochemistry

Immunoprecipitation (IP), immunoblotting (IB), confocal microscopy, and immunohistochemistry (IHC) assays were carried out as previously described [22, 23]. 40 glioma samples for IHC analysis were collected from the Second Affiliated Hospital of Dalian Medical University (Dalian, China). The study was performed with approval from the Ethics Committee at the Dalian Medical University. Written informed consent was obtained from all patients and data were analyzed anonymously. Anti-FKBP9 (1:100), anti-Ki67 (1:400), anti-pIRE1α (1:200) antibodies were used for IHC.

### Quantitative real time PCR (qRT-PCR)

With SYBR Select Master Mix (Applied Biosystems, USA), mRNA levels of genes were analyzed in the Mx3005P Real-Time PCR system (Aglient, USA). The relative transcription levels of the genes were calculated using the delta-delta-Ct (ΔΔCT) method (expressed as 2 − ΔΔCT) and normalized to GAPDH as an endogenous control. Primers are shown as follows:

*fkbp9*: F- GAAAAGCGAAGGATTGTGGTC

R- TTTGTAGTGGGAGGTGATGC

*fkbp9(exon9)*: F- GAATGGAGATGGGAAGGTCAC

R- CCCTCACTGCACGTCTTG

*chac1*:F- GCCCTGTGGATTTTCGGGTA

R- TGTGGGATTGAGGGTCACATC

*ddit3*: F- GGAGCATCAGTCCCCCACTT

R- TGTGGGATTGAGGGTCACATC

*il6*: F- TCCATCCAGTTGCCTTCTTG

R- ATTGCCATTGCACAACTCTTTT

*sel1l*: F- ATCTCCAAAAGGCAGCAAGC

R- TGGGAGAGCCTTCCTCAGTC

*gapdh*:F- CTTCACCACCATGGAGGAGGC

R- GGCATGGACTGTGGTCATGAG

### In vivo xenograft model

For subcutaneous tumor formation, SF-539 cells (2 × 10^6^) were injected subcutaneously into backs of nude mice (5–6 weeks age, Beijing Vital River Laboratory Animal Technology Co., Ltd). Tumor diameters were measured every three days and the tumor volume calculated using the formula: V = 0.52 × L × W^2^. For establish a GBM orthotopic mouse model, 1 × 10^5^ LN-229-shFKBP9 or control cells were stereotactically implanted into the right caudate nucleus of the nude mice (depth 3.5 mm). Tumor-bearing mice were sacrificed with ether anesthesia, and bioeluminance imaging was performed at day 28.

All Animal experiments complied with the national guidelines for the care and use of laboratory animals. Procedures involving mice, approved by the experimental animal ethics committee, were operated at Dalian Medical University Laboratory Animal Center.

### Statistical analysis

All data in this experiment were performed by GraphPad Prism 7 software. Double-tailed t-test or one-way ANOVA were used to analyzed the difference between data. The results were shown as mean ± standard error. Significant difference: *p < 0.05, **p < 0.01, ***p < 0.001.

## Results

### FKBP9 is upregulated in human high-grade gliomas and correlates with poor prognosis

**Fig. 1:**
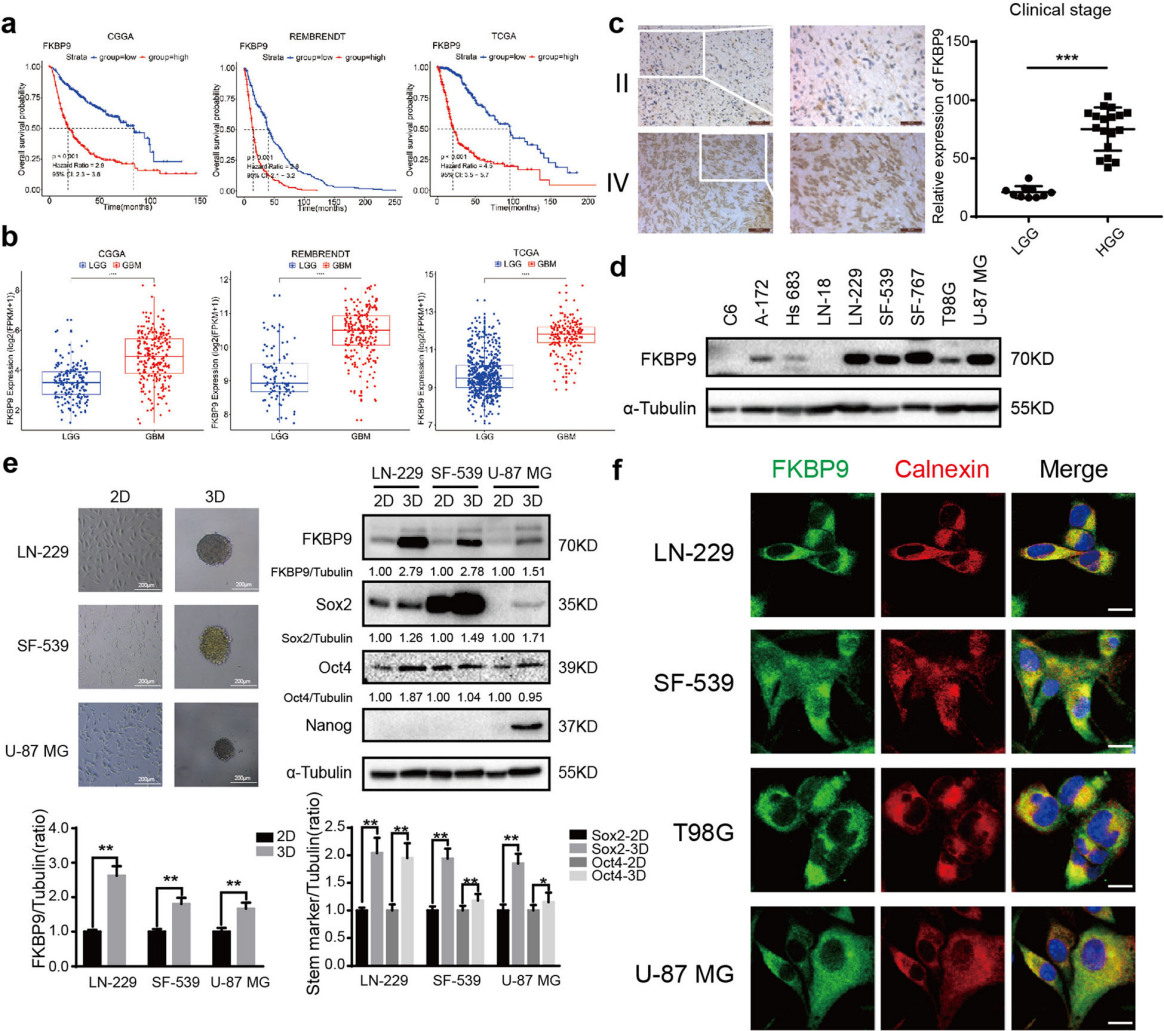
FKBP9 is elevated in high grade gliomas and correlates with poor prognosis. **a** Kaplan–Meier survival analysis of glioma samples from CGGA (*n* = 702), REMBRENDT (*n* = 619) and TCGA (*n* = 476) data, respectively (all *p* < 0.001). **b** Comparison of FKBP9 mRNA expression between low-grade gliomas (LGGs) and high-grade gliomas (HGGs) from the three databases used in (**a**). **c** Immunohistochemistry (IHC) analysis of FKBP9 on 40 specimens including low and high grade gliomas. Scale bar = 100 μm. Score according to the degree of cell staining and the proportion of positive cells. The protein expression level was shown as the product value of two scores (**p < 0.01). **d** Immunoblotting (IB) analysis for FKBP9 protein levels in GBM cell lines. α-Tubulin was used as a loading control. **e** Images for spheres of LN229, SF-539 and U-87 MG cells within 10 days of three dimensional (3D) culture. Scale bar = 200 μm (× 10). IB analysis for FKBP9, Sox2, Oct4 and Nanog protein levels in 2D and 3D cultured cells. α-Tubulin was used as a loading control. The ratios of FKBP9, Sox2 and Oct4 expression to their corresponding α-Tubulin were represented. **f** LN229, SF-539, T98G and U-87 MG cells were fixed for immunofluorescence (IF) and stained for FKBP9 (green), Calnexin (red) and DAPI (blue). Representative merged images were also shown for fluorescence signals. Scale bar = 25 μm. All experiments in this figure were performed three times with comparable results

Our initial analysis of FKBP9 expression using TCGA by cBioPortal for Cancer Genomics (http://www.cbioportal.org/) revealed that the FKBP9 gene was highly amplified in gliomas across all cancer types (Additional file 1: Figure S1a). To evaluate the relevance of FKBP9 to glioma prognosis, three datasets from the Chinese Glioma Genome Atlas (CGGA), the Repository for the Molecular Brain Neoplasia Data (REMBRANDT) of the National Cancer Institute and TCGA (The Cancer Genome Atlas) databases were examined by Kaplan–Meier survival analysis based on FKBP9 mRNA expression. The results showed that high FKBP9 expression correlated with reduced overall survival (OS) of glioma patients (Fig. 1a). Notably, higher FKBP9 expression was also observed in GBM compared with low-grade glioma (LGG, WHO grade I and II gliomas) (Fig. 1b). To further investigate the correlation between FKBP9 and GBM, immunohistochemical (IHC) analysis of the FKBP9 protein was performed on paraformaldehyde-fixed tissue sections from 40 patient-derived glioma samples (Additional file 7: Table S1). Abundant FKBP9 staining was detected in the cytoplasm of GBM tissues (*n* = 19) but much less in LGG tissues (*n* = 16) (Fig. 1c, representative results shown). In addition, the FKBP9 protein was expressed in nine established GBM cell lines (Fig. 1d). Notably, under three-dimensional (3D) culture conditions, FKBP9 levels positively correlated with the levels of the stem markers Oct4 and Sox2 in LN-229- and SF-539-derived spheres as well as Nanog in U-87 MG-derived spheres (Fig. 1e and Additional file 1: Figure S1b). Accordingly, the strong positive correlation between the expression levels of FKBP9 and the stem markers Sox2, Oct4 and Nestin, was demonstrated in at least one of the three datasets (TCGA, CGGA and REMBRANDT) (Additional file 1: Figure S1c). In addition, endogenous FKBP9 predominantly colocalized with the ER marker Calnexin in LN-229, SF-539, T98G and U-87 MG cells (Fig. 1f). Consistent with these findings, most of the ectopic V5-tagged FKBP9 resided in the ER in T98G and U-87 MG cells, while an FKBP9 mutant in which the C-terminal ER resident motif (KDEL) was deleted localized to much lower extent to the ER than did wild type FKBP9 (Additional file 1: Figure S1d).

### Depletion of FKBP9 suppresses malignant phenotypes of GBM cells in vitro

**Fig. 2:**
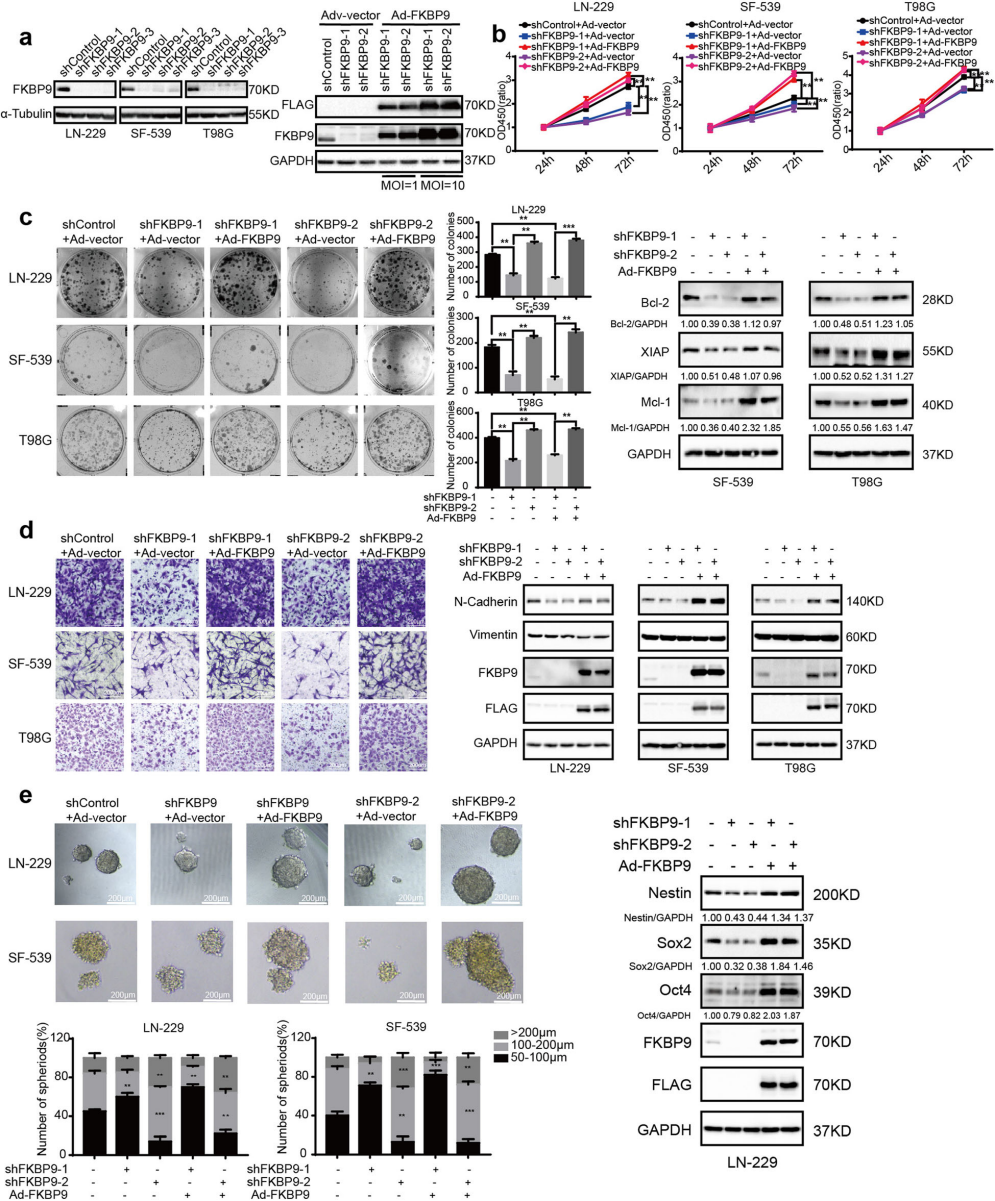
FKBP9 depletion inhibits the malignant phenotypes of GBM cells in vitro. **a** Efficiencies for stable knockdown in LN-229, SF-539 and T98G cells of FKBP9 (indicated as LN-229-shControl, LN229-shFKBP9; SF-539-shControl, SF-539-shFKBP9; T98G-shControl, T98G-shFKBP9) were tested by IB assays. IB analysis for the rescue efficiency of adenoviruses overexpressing FKBP9 in T98G-shFKBP9 cells using GAPDH as a loading control (MOI = 1 or 10). **b-d** LN-229-shFKBP9, SF-539-shFKBP9 and T98G-shFKBP9 cells were introduced with adenoviruses carrying the vector control (Ad-vector) and adenoviruses overexpressing FKBP9 (Ad-FKBP9). Analysis of cell viability, colony formation and invasion of these cells was performed. Protein levels of Bcl-2, XIAP and Mcl-1, N-Cadherin and Vimentin were detected by IB assays. **e** Analysis of the ability of LN-229-shFKBP9 and SF-539-shFKBP9 cells with or without Ad-FKBP9 to form spheres in 3D cultures. Scale bar = 200 μm (× 10). Number and size of spheres were counted and measured. Stem markers including Sox2 and Oct4 of LN-229 cells spheres were detected by IB assays. All experiments were performed three times with comparable results. Data are represented as mean ± S.D. (*p < 0.05, **p < 0.01, ***p < 0.001)

To examine the in vitro function of FKBP9 in GBM, we stably introduced lentiviral vectors containing three distinct shRNAs specifically targeting FKBP9 or nontargeting control shRNAs into the LN-229, SF-539 and T98G GBM cell lines (designated LN-229-shFKBP9, SF-539-shFKBP9, T98G-shFKBP9, LN-229-shControl, SF-539-shControl and T98G-shControl, respectively), and the knockdown efficiency was confirmed by immunoblotting (IB) assay (Fig. 2a, left panel). In addition, adenovirus-mediated expression of FLAG-tagged FKBP9 or vector (designated Ad-FKBP9 and Ad-vector, respectively) was used to restore FKBP9 expression in GBM cells with FKBP9 knockdown (Fig. 2a, right panel). We then performed a set of cell-based assays, including CCK-8 and colony formation assays, to dissect the biological functions of FKBP9. As shown in Fig. 2b and c, depletion of FKBP9 significantly decreased cell proliferation and colony formation in LN-229-shFKBP9, SF-539-shFKBP9 and T98G-shFKBP9 cells compared to their corresponding control cells. In addition, we observed that several pro-survival proteins, including Bcl-2, XIAP and Mcl-1, were substantially downregulated in SF-539-shFKBP9 and T98G-shFKBP9 cells (Fig. 2c, Additional file 2: Figure S2a and S2b). Rescue experiments in which FKBP9 knockdown cells were infected with Ad-FKBP9 revealed that the decreased proliferation, colony formation and downregulated pro-survival protein levels in FKBP9-depleted GBM cells were a consequence of FKBP9 deficiency (Fig. 2b, c and Additional file 2: Figure S2a, S2b). Moreover, transwell invasion assays showed that LN-229-shFKBP9, SF-539-shFKBP9 and T98G-shFKBP9 cells demonstrated a significantly reduced capacity to invade compared to the control cells, with decreased expression levels of N-Cadherin in the FKBP9-depleted GBM cells (Fig. 2d). As expected, Ad-FKBP9 rescued the above effects in GBM cells with FKBP9 depletion (Fig. 2d). Furthermore, FKBP9 depletion significantly reduced sphere formation in LN-229 and SF-539 GBM cells, which was rescued by the introduction of Ad-FKBP9 (Fig. 2e). Knockdown of FKBP9 also led to the downregulation of the stem cell markers Oct4 and Sox2 in LN-229-derived spheres, which was reversed by Ad-FKBP9 (Fig. 2e).

### FKBP9 depletion inhibits GBM growth in vivo

**Fig. 3:**
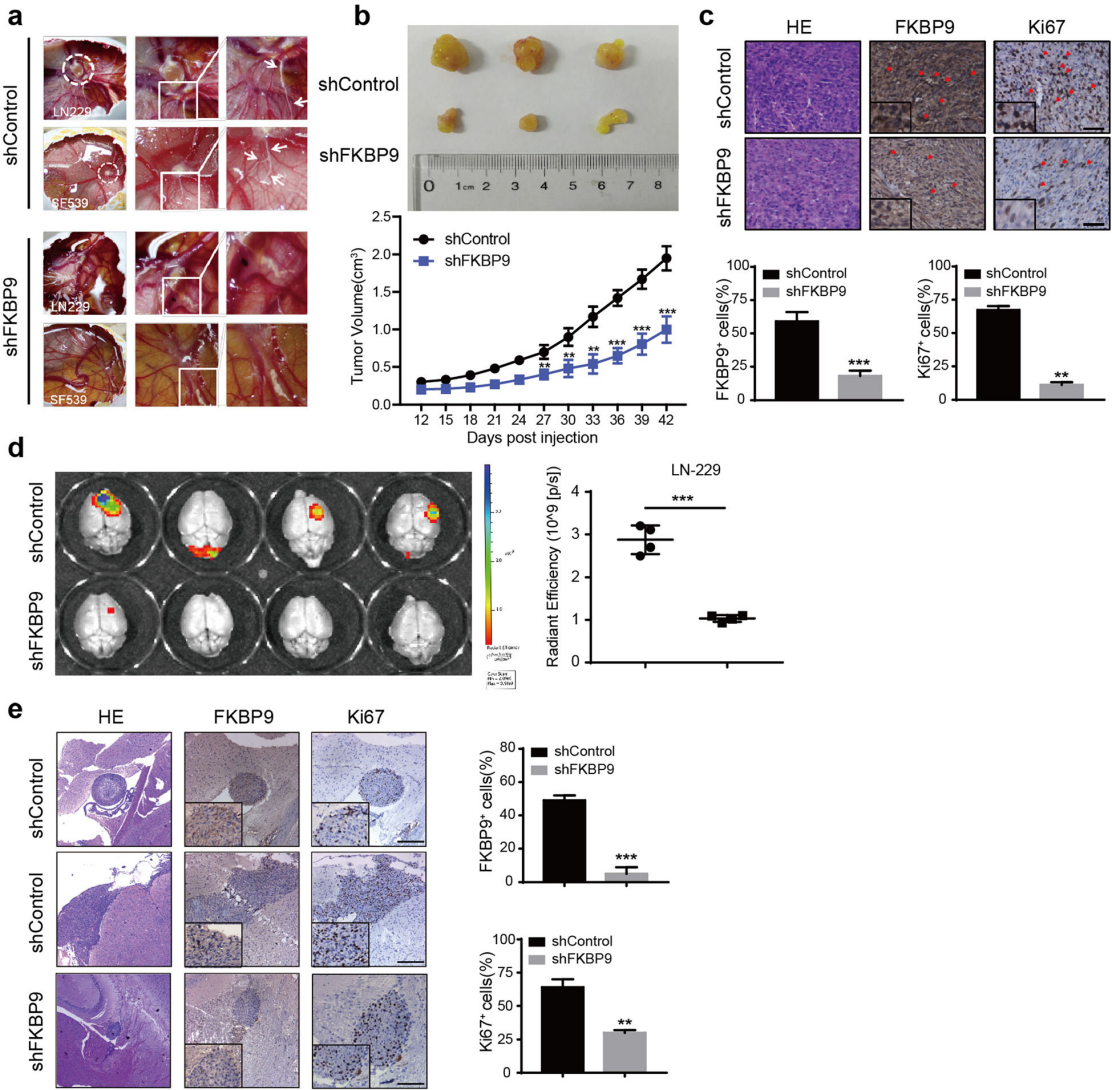
FKBP9 depletion suppresses GBM growth in vivo. **a** Images of chick embryo’s chorioallantoic membrane (CAM) tumors in shControl and shFKBP9 cells 7 days after implantation. LN229-shControl and LN229-shFKBP9 cells (n = 4 eggs for each group), SF-539-shControl and SF-539-shFKBP9 cells (*n* = 5 eggs for each group) were digested and implanted as an amount of 1.0 × 10^6^ per egg. Tumors were circled with white dotted lines, and the areas marked with squares were enlarged to show cell invasion along blood vessels which was indicated with arrows. **b** Representative images of tumors derived from SF-539-shControl (n = 5 mice) and SF-539-shFKBP9 cells (n = 5 mice) 45 days post injection. Tumor volumes were measured every three days. **c** HE staining was performed in SF-539-shControl and SF-539-shFKBP9 tumors, and the expression of FKBP9 and Ki67 were detected by IHC assays. Scale bar = 50 μm. Magnified insets showed FKBP9 or Ki67 positive stained cells. **d** Intracranial injection of LN-229 cells with or without FKBP9 depletion into nude mice (*n* = 8). Bioluminescent images and quantification of xenografts derived from LN-229 implantation were shown. **e** Representative images of HE and tumor sections staining with FKBP9 and Ki67 by IHC were shown. (**p < 0.01, ***p < 0.001)

The in vivo effects of FKBP9 knockdown on GBM cells were first examined in a chick embryo chorioallantoic membrane (CAM) model. As shown in Fig. 3a, depletion of FKBP9 in SF-539 cells led to a substantial decrease in both tumor growth and vascular invasion in the CAM model compared to the control. To investigate the role of FKBP9 in GBM growth in vivo, we implanted SF-539-shControl and SF-539-shFKBP9 cells into immunodeficient nude mice. Consistent with our findings in the CAM model, the tumors generated by SF-539-shFKBP9 cells grew smaller than those derived from the control cells (Fig. 3b). Ki67 staining confirmed decreased proliferation in tumors with FKBP9 depletion (Fig. 3c).

To further explore the effects of FKBP9 expression on glioblastoma progression in vivo, we performed orthotopic mouse xenograft experiments. The results indicated that the volumes of tumors derived from FKBP9-depleted LN-229 cells were much lower than those of control tumors (Fig. 3d). Moreover, tumors derived from shControl cells spread widely in the mice brain besides primary location compared to that derived from shFKBP9 cells. Similarly, we observed decreased Ki67 staining in FKBP9-deficient tumors (Fig. 3e).

### The FKBP9 M541I variant exhibits less oncogenic effects than its wild-type counterpart

**Fig. 4:**
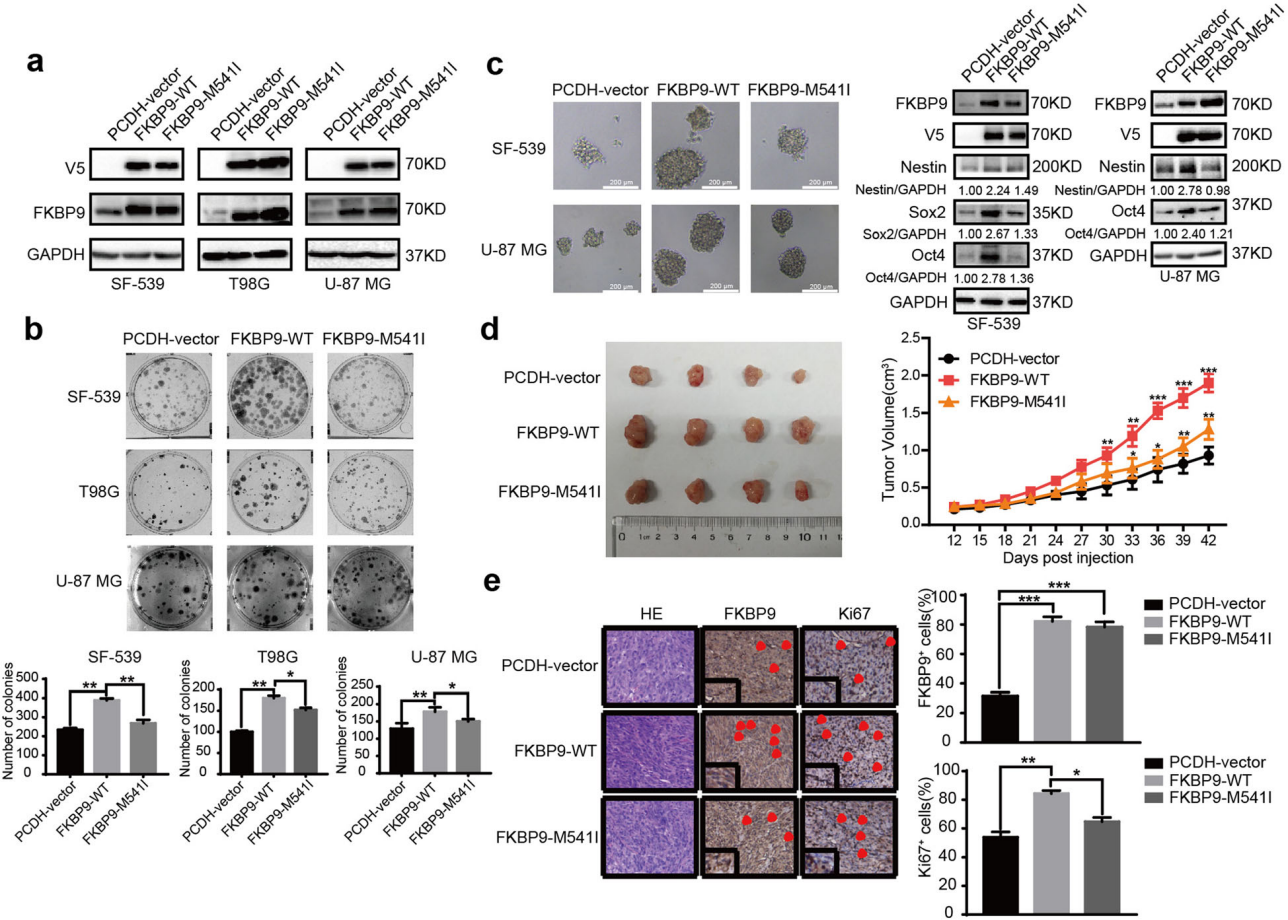
FKBP9-M541I mutant displays a weaker carcinogenesis compared with the wide type counterpart. **a** Efficiencies for stable overexpresssion of FKBP9 in SF-539, T98G and U-87 MG cells of FKBP9 (indicated as PCDH-vector, FKBP9-WT and FKBP9-M541I) were tested by IB assay. **b**, **c** Analysis of the ability of FKBP9-WT- and FKBP9-M541I-overexpressed SF-539, T98G and/or U-87 MG cells to form colonies and spheres. Expression of Nestin, Sox2 and/or Oct4 were detected by IB. **d** Images of mice tumors from SF-539-PCDH-vector, SF-539-FKBP9 and SF-539-M541I cells 45 days post injection. (n = 5 mice for each). Tumor volumes were measured. **e** HE staining and IHC analysis of FKBP9 and Ki67 expression were performed. Scale bar = 50 μm. (*p < 0.05, **p < 0.01, ***p < 0.001)

A previous study by Verhaak and colleagues reported a mutation of methionine (M) at position 541 into isoleucine (I) in FKBP9 in clinical glioma tissue samples [13]. We wanted to investigate the biological significance of this mutation in FKBP9. We stably introduced wild-type (WT)-FKBP9 or the FKBP9-M541I variant into SF-539, T98G and U-87 MG GBM cells (designated SF-539-FKBP9-WT, SF-539-FKBP9-M541I, T98G-FKBP9-WT, T98G-FKBP9-M541I, U-87 MG-FKBP9-WT and U-87 MG-FKBP9-M541I respectively) and confirmed expression of the constructs by IB (Fig. 4a). Figure 4b and c show that ectopic expression of the FKBP9 M541I mutant in GBM cells did not alter the colony/sphere formation potential relative to that in control cells. Consistent with this result, spheres derived from two GBM cell lines overexpressing the FKBP9-M541I variant did not exhibit upregulation of the stem cell markers Nestin, Sox2 and/or Oct4 as observed in wt-FKBP9-overexpressing spheres (Fig. 4c). The in vivo effects of the FKBP9-M541I variant on GBM growth were evaluated in a mouse xenograft model. As shown in Fig. 4d, mice engrafted with SF-539 cells expressing the FKBP9-M541I variant had smaller tumors than those engrafted with wt-FKBP9-overexpressing cells. Ki67 staining results showed decreased proliferation in tumors with FKBP9-M541I compared to tumors with wt-FKBP9 (Fig. 4e).

### p38MAPK is critical for FKBP9-driven oncogenic activity

**Fig. 5:**
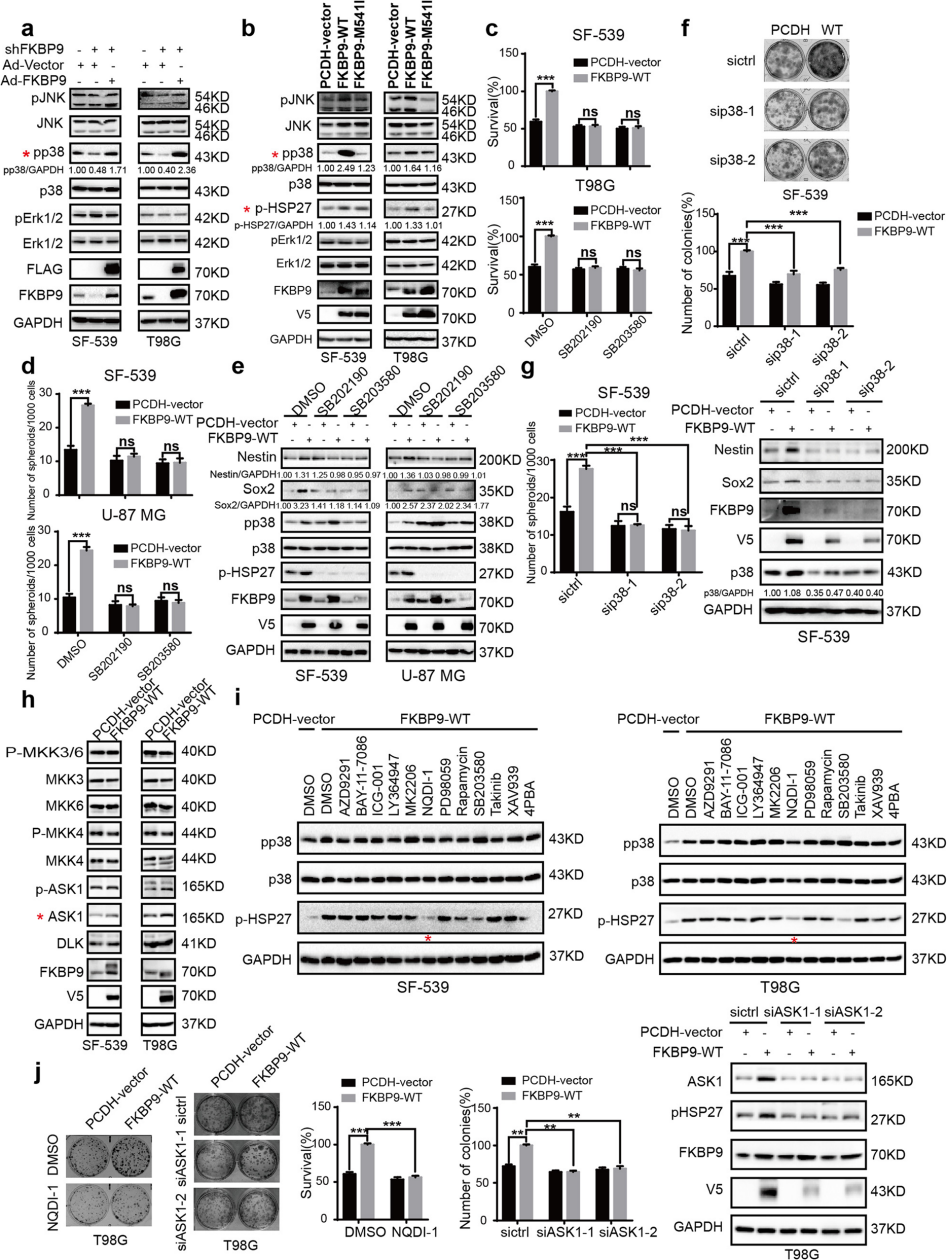
p38MAPK contributes to FKBP9’s oncogenic function in GBM cells. **a** Cell lysates from SF-539 and T98G cells treated as in Fig. 2b were analyzed by IB for key protein levels of MAPK pathway. **b** Protein levels of p38 and its downstream genes in SF-539-FKBP9-WT/FKBP9-M541I and T98G-FKBP9-WT/FKBP9-M541I cells were detected by IB. **c, d** SF-539-FKBP9-WT and T98G-FKBP9-WT cells were treated with vehicle or 5 μM SB201290/SB203580 and the ability of these cells to form colonies and spheres was determined. **e** Nestin and Sox2 levels of SF-539 and U-87 MG cells spheres were detected by IB. **f, g** SF-539-PCDH-vector and FKBP9-WT cells were transfected with two siRNA duplexes targeting p38 (sip38) or control siRNA (siCtrl) for 48 h. Colony formation and sphere formation assays were performed. Nestin and Sox2 expression were detected by IB. **h** Protein expression of p38’s upstream regulators in SF-539-FKBP9-WT and T98G-FKBP9-WT cells was detected by IB. **i** SF-539-FKBP9-WT and T98G-FKBP9-WT cells were treated with vehicle (DMSO), AZD9291 (1 μM), BAY-11-7086 (10 μM), ICG-001 (5 μM), LY364947 (10 μM), MK2206 (2 μM), PD98059 (10 μM), NQDI-1 (5 μM), Rapamycin (2 μM), SB203580 (2 μM), Takinib (5 μM), XAV939 (10 μM) or 4PBA(50 μM), respectively. IB analysis was performed for p-p38, p38 and pHSP27. GAPDH was used as a loading control. **j** Cells transfected with PCDH-vector or FKBP9-WT were treated with vehicle or 5 μM NQDI-1, or transfected with two siRNA duplexes targeting ASK1 (siASK1) or control siRNA (siCtrl) for 48 h. Colony formation assays were performed. All experiments in this figure were performed three times with comparable results. Data are represented as mean ± S.D. (**p < 0.01, ***p < 0.001)

Next, we explored the mechanism(s) underlying FKBP9-mediated oncogenic activity in GBM cells. By analyzing several key cell growth-related pathways such as the MAPK (ERK1/2, JNK, and p38MAPK), PI3K-AKT-mTOR, WNT, JAK–STAT, Hippo and NF-κB signaling pathways, we observed that stable FKBP9 knockdown led to a remarkable decrease in the phosphorylation levels of p38MAPK and a reduction in cyclinD1 levels in both SF-539 and T98G cells (Fig. 5a and Additional file 3: Figure S3a), which could be rescued by Ad-FKBP9. In contrast, stable FKBP9 expression resulted in robust activation of p38MAPK as well as an increase in the phosphorylation levels of pS6 and p4EBP1 in SF-539 cells and a modest increase in T98G cells (Fig. 5b and Additional file 3: Figure S3b). However, stable overexpression of FKBP9-M541I failed to effectively activate p38MAPK as did its wild-type counterpart (Fig. 5b). FKBP9-regulated activation of p38MAPK was further demonstrated by detecting the change in the phosphorylation levels of HSP27, a putative p38MAPK substrate (Fig. 5b). We next examined whether p38MAPK is responsible for the FKBP9-mediated oncogenic effects in GBM cells. As shown in Fig. 5c, treatment with two p38MAPK inhibitors SB202190 and SB203580 significantly impaired FKBP9 overexpression-induced colony formation in both SF-539 and T98G cells. In addition, both inhibitors significantly reduced the formation of spheres in FKBP9-overexpressing SF-539 and U-87 MG cells (Fig. 5d). Similarly, we observed the downregulation of the stemness markers Nestin and Sox2 in these cells upon exposure to the p38MAPK inhibitors (Fig. 5e). The effects of these inhibitors on p38MAPK signaling were evaluated by phosphorylation of HSP27 (Fig. 5e).

To rule out the possible off-target effects of the p38MAPK inhibitors, we knocked down p38MAPK with 2 different siRNAs in SF-539-FKBP9-WT cells and confirmed the expression of p38MAPK (Fig. 5f). Knockdown of p38MAPK significantly attenuated FKBP9 overexpression-induced colony and sphere formation in SF-539-FKBP9-WT cells compared to the control siRNA treatment (Fig. 5f and g). Moreover, the protein levels of Nestin and Sox2 in SF-539-FKBP9-WT cells were dramatically reduced upon p38MAPK knockdown (Fig. 5g).

To explore how FKBP9 expression regulates p38MAPK activation, we investigated the activation and expression of the potential signaling molecules upstream of p38MAPK signaling, which included MKK3/6, MKK4, ASK1 and DLK. The results indicated that ASK1 levels were markedly upregulated in FKBP9-overexpressing SF539 and T98G cells whereas activation of ASK1 (ASK1 phosphorylation at Ser 83) was not altered (Fig. 5h). In addition, there was no alteration in the activation or expression levels of other signal molecules upstream of p38MAPK between FKBP9-overexpressing cells and their control parental cells (Fig. 5h). Furthermore, both NQDI-1, a known ASK1 inhibitor, and siRNA-mediated knockdown of ASK1, substantially attenuated the increased phosphorylation of HSP27 (indicator of p38 activation) in FKBP9-overexpressing SF-539 and T98G cells (Fig. 5i, j and Additional file 3: Figure S3c), indicating that FKBP9 expression induced the activation of p38MAPK in GBM cells at least in part through ASK1. Consistent with these findings, we observed that both siRNA-mediated knockdown of ASK1 and exposure to NQDI-1 (ASK1 inhibitor) significantly impaired the increased clonogenic growth potential of FKBP9-overexpressing SF-539 and T98G cells (Fig. 5j and Additional file 3: Figure S3c), suggesting that ASK1 could play a role in the FKBP9-mediated effects on GBM cell growth.

### Depletion of FKBP9 activates the IRE1α-XBP1 pathway

**Fig. 6:**
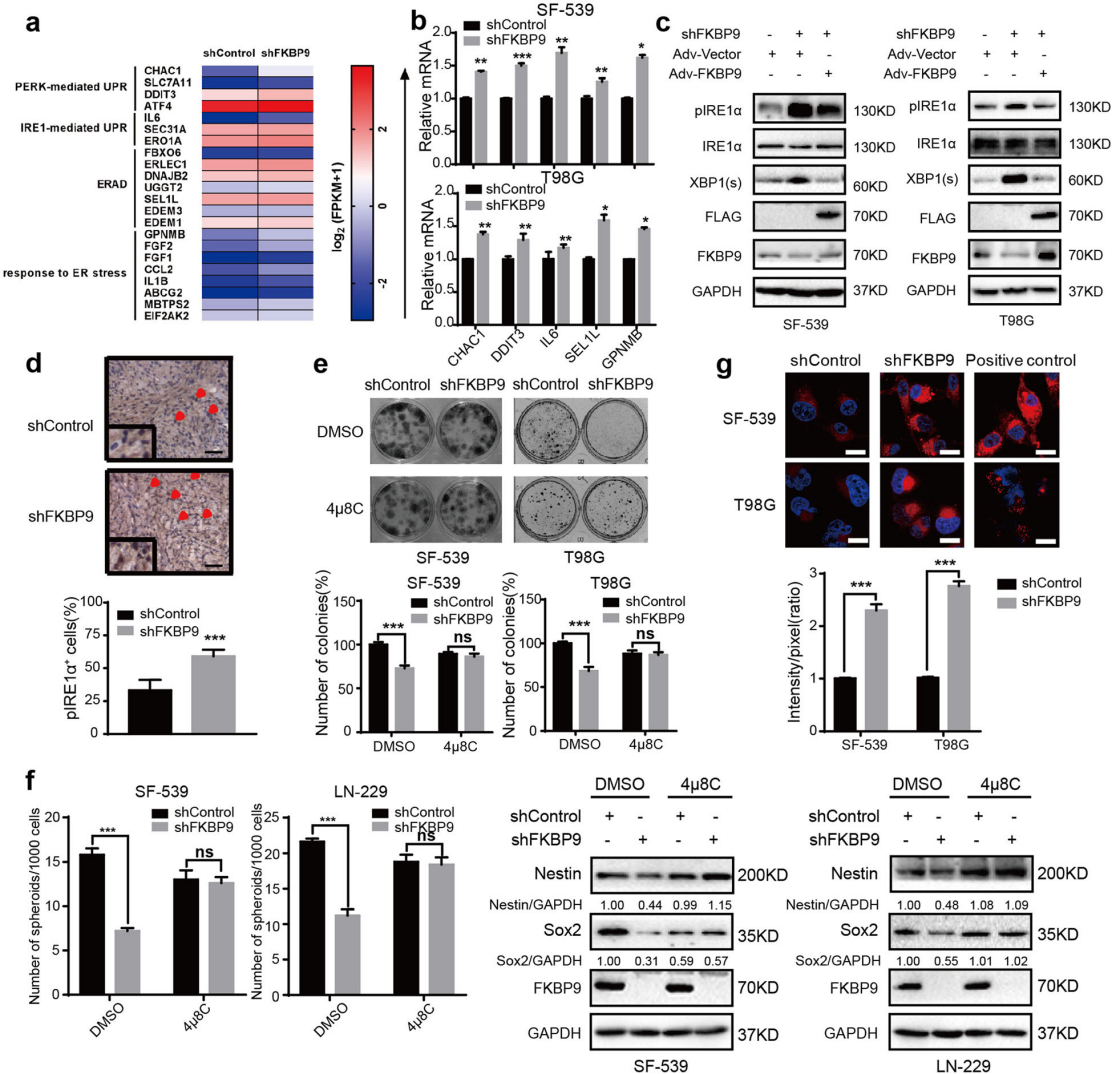
Knockdown FKBP9 activates the IRE1α-XBP1 pathway. **a** The list of 22 significant transcripts that are regulated by FKBP9 deficiency were classified into 4 categories including PERK-mediated UPR, IRE1-mediated UPR, ERAD and other proteins response to ER stress (Additional file 9: Table S3). **b** Real-time quantitative PCR analysis for CHAC1, DDIT3, IL6, SEL1L and GPNMB mRNA levels of stable FKBP9-depleted SF-539 and T98G cells. **c** IB analysis for pIRE1α and XBP1(s) expression in stable FKBP9-depleted and adenovirus-overexpressed SF-539 and T98G cells treated as in Fig. 2a. GAPDH was used as a loading control. **d** Tumor tissue sections from SF-539-shControl and SF539-shFKBP9 cells were analyzed by IHC using anti-pIRE1α antibody. Scale bar = 50 μm. Magnified insets showed pIRE1α positive stained cells. **e, f** Colony formation and sphere formation assays were performed in stable FKBP9-depleted SF-539, T98G or U-87 MG cells treated with vehicle or 50 μM 4μ8C. Nestin and Sox2 expression of SF-539 and U-87 MG cells spheres were detected by IB. **g** Aggresomes in stable FKBP9-depleted SF-539 and T98 cells were analyzed using Aggresome Detection kit by confocal microscopy. MG132 (5 μM for 8 h) was used as a positive control. Fluorescence intensity was quantified by Image J. Data are presented as mean ± SEM from three independent experiments. (*p < 0.05, **p < 0.01, ***p < 0.001)

Our data in Fig. 1g show that FKBP9 localized to the ER in GBM cells, thus, we hypothesized that FKBP9 could regulate ER function such as ER stress or the UPR pathway. To test this hypothesis, we performed microarray analysis on SF-539 cells with or without FKBP9 knockdown. Depletion of FKBP9 upregulated a total of 144 genes and downregulated 158 genes (*p* < 0.05, log2 fold change> 1) in SF539-shFKBP9 cells (Additional file 8: Table S2). Among these upregulated genes, 22 genes were involved in ER stress or UPR signaling (Fig. 6a). Changes in the upregulated genes associated with ER stress or UPR signaling in SF539-shFKBP9 cells were further confirmed by RT-qPCR (Fig. 6b). We also validated the changes in these genes in T98G-shFKBP9 cells (Fig. 6b). We next examined whether FKBP9 perturbs the UPR pathway. As shown in Fig. 6c, stable knockdown of FKBP9 in SF-539 and T98G GBM cells led to the activation of IRE1α and XBP1 splicing, which was partially rescued by Ad-FKBP9. Accordingly, our RT-PCR analysis indicated the upregulation of IL6, a known downstream target gene regulated by the IRE1α-XBP1 pathway, in SF-539-shFKBP9 and T98G-shFKBP9 cells (Fig. 6b). Changes in other branches of UPR signaling, i.e., PERK and ATF6, were not consistent among the tested GBM cell lines upon FKBP9 depletion (data not shown). To explore whether the IRE1α-XBP1 pathway plays a role in FKBP9-mediated oncogenic activities in GBM cells, we treated the cells with 4μ8C, a specific inhibitor of IRE1α activity. As shown in Fig. 6e, 4μ8C treatment prevented the FKBP9 depletion-mediated decrease in colony formation in SF-539 and T98G cells. Exposure to 4μ8C also reversed the decrease in sphere formation in LN-229 and SF-539 cells induced by FKBP9 knockdown (Fig. 6f). Consistent with the above in vitro results, we detected a significant increase in pIRE1-α staining by IHC in tumors derived from SF-539-shFKBP9 cells compared to SF-539-shControl cells (Fig. 6d).

UPR signaling is often activated upon ER stress, which is characterized by disrupted protein folding and accumulation of misfolded/unfolded proteins (aggregated proteins), which can be determined by an aggresome formation assay [24]. We then examined whether the FKBP9 depletion-induced activation of UPR signaling is due to protein misfolding and accumulation. As shown in Fig. 6g, depletion of FKBP9 led to aggresome formation in both SF-539 and T98G cells as indicated by cellular aggresome visualization using an Aggresome Detection kit, but not in control cells. In addition, knockdown of p38MAPK did not affect the downregulation of pIRE1α levels in SF-539-FKBP9 and T98G-FKBP9 cells (Additional file 4: Figure S4a). Taken together, our data suggest that the IRE1α-XBP1 pathway is, at least partly, associated with growth inhibition induced by FKBP9 knockdown in GBM cells.

### FKBP9 expression confers GBM cell resistance to ER stress inducers

**Fig. 7:**
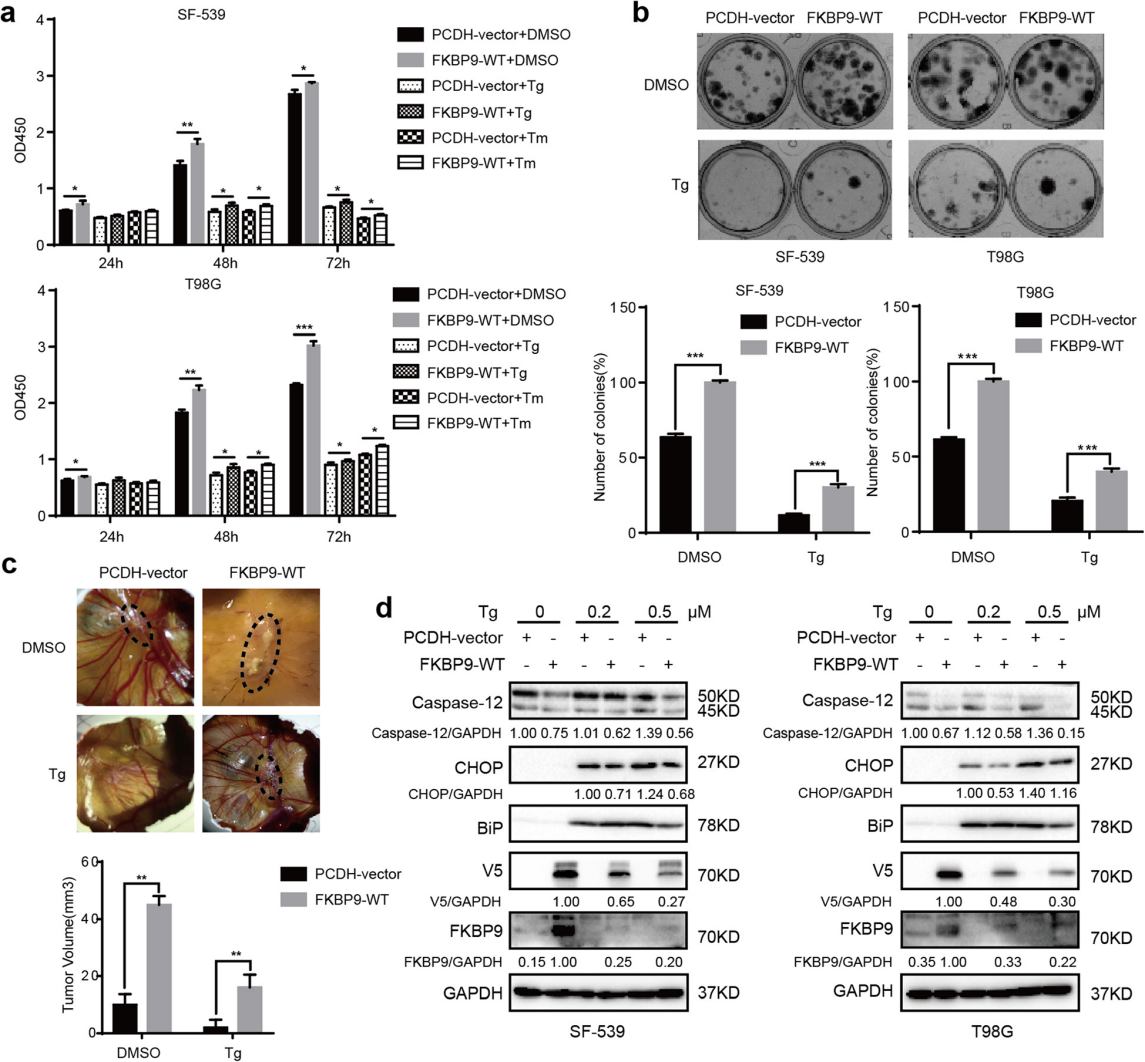
FKBP9 expression resists to ER stress inducer-triggered cell death. **a** Cell viability of SF-539-FKBP9 and T98G-FKBP9 cells exposed to Tg (0.2 μM) or Tm (1.2 μM) for 24, 48 and 72 h were measured by Cell Counting Kit-8. **b** Analysis of colony formation of SF-539-FKBP9 and T98G-FKBP9 cells treated with vehicle and 0.1 μM Tg for 12 days. **c** SF-539-PCDH-vector and SF-539-FKBP9-WT cells were pretreated with Tg (0.5 μM) for 6 h, then implanted into CAM (5 eggs for each). Representative images for tumors after 7 days were shown. **d** IB analysis for Caspase-12 and CHOP expression in SF-539-FKBP9-WT and T98G-FKBP9-WT cells exposed with Tg for 12 h. The ratios of Cleaved-Caspase-12 and CHOP expression to their corresponding GAPDH were represented. Data are represented as mean ± SEM from three independent experiments, *p < 0.05, **p < 0.01, ***p < 0.001

Induced imbalance in ER proteostasis in GBM has been employed as a therapeutic approach. Our above data indicated that depletion of FKBP9 upregulated UPR signaling by inducing ER stress. We then examined whether FKBP9 expression could confer resistance of GBM cells to ER stress inducer-triggered cell death. Both thapsigargin (Tg) and tunicamycin (Tm), two ER stress inducers, time-dependently activated UPR signaling and induced the expression of the ER stress markers BiP and CHOP in both SF-539 and T98G GBM cells (Additional file 5: Figure S5a). We observed that treatment of SF-539-FKBP9-WT and T98G-FKBP9-WT cells with either Tg or Tm led to a significant reduction in cell death (Fig. 7a) and colony formation (Fig. 7b and Additional file 5: Figure S5b) when compared to similarly treated control cells. In contrast, Tg or Tm treatment resulted in more cell death and less colony formation in SF-539-shFKBP9 and T98G-shFKBP9 cells than in control cells (Additional file 5: Figure S5d). Similarly, stable expression of FKBP9 rendered SF-539 cells resistant to Tg in the CAM model (Fig. 7c). Furthermore, Tg or Tm treatment elicited a dramatic decrease in the levels of BiP, CHOP and cleaved Caspase-12 in SF-539-FKBP9-WT and T98G-FKBP9-WT cells compared to similarly treated control cells (Fig. 7d and Additional file 4: Figure S4b). In addition, we observed that the M541I mutation in FKBP9 failed to confer GBM cell resistance to ER stress inducers (Additional file 5: Figure S5c).

### FKBP9 is degraded during Tg-induced stress

**Fig. 8:**
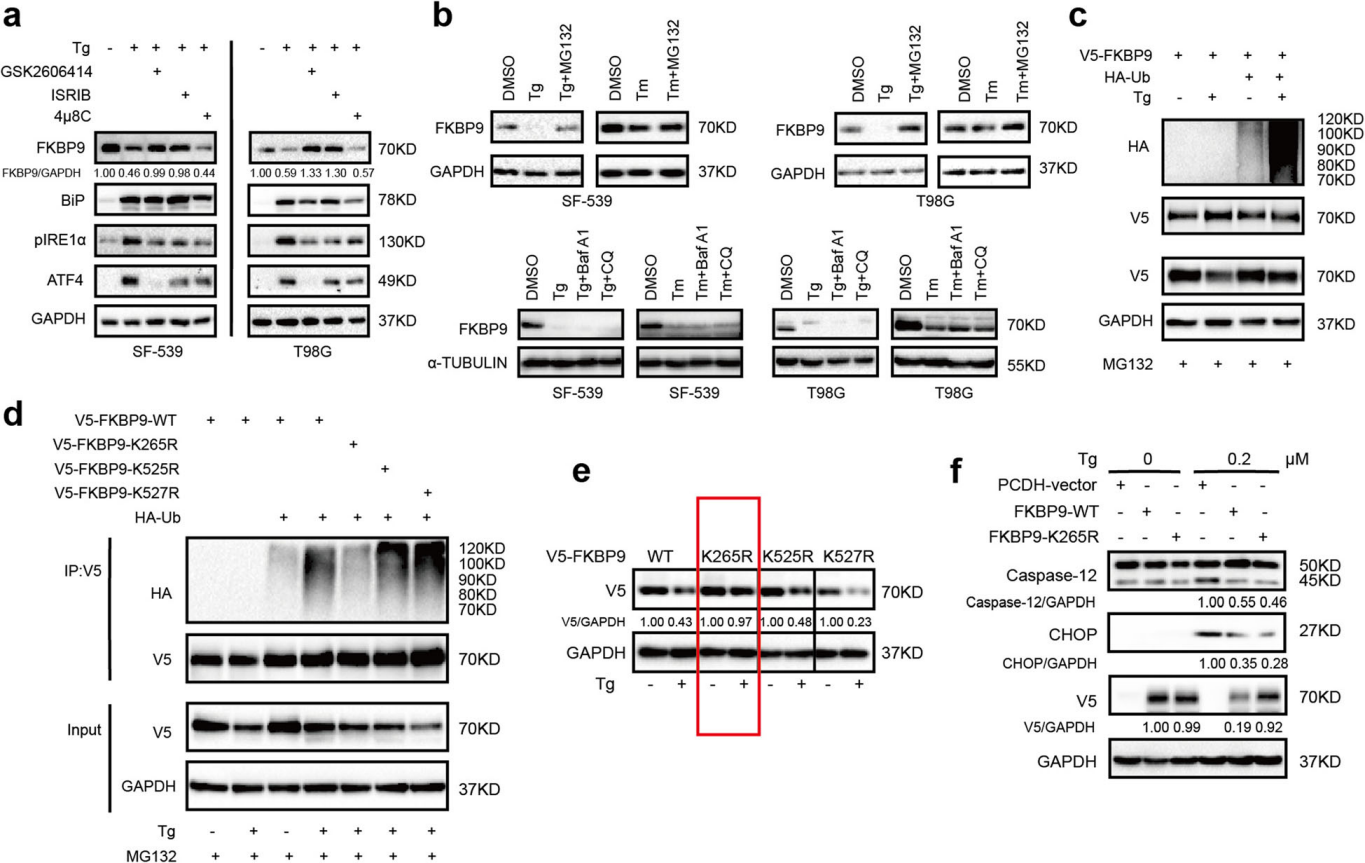
FKBP9 is degraded during Tg-induced ER stress. **a** SF-539 and T98G cells were pretreated with vehicle, GSK260414 (1 μM), ISRIB (100 nM) or 4μ8C (50 μM), and then exposed to vehicle, 0.5 μM Tg or 2.5 μM Tm for 6 h. IB analysis for FKBP9 and protein levels in UPR. **b** SF-539 and T98G cells were treated with vehicle, 0.5 μM Tg or 2.5 μM Tm for 6 h, and MG132 (1 μM), Baf A1 (10 nM), CQ (5 μM) were added to the cells 1 h in advance. Expression of FKBP9 was tested by IB using GAPDH as a loading control. **c** HEK-293 T cells were transfected with V5-tagged FKBP9, HA-tagged Ub, and then they were treated with Tg for 6 h. Cells were pretreated with MG132 the same as in (**b**). Immunoprecipitation (IP) was performed with anti-V5 antibody, IB with the indicated antibodies. **d** HEK-293 T cells were tranfected with HA-tagged Ub, V5-tagged FKBP9 wide-type and mutants, and then they were treated with Tg for 6 h. Similar analysis were performed as in (**c**) for FKBP9 ubiquitination. **e** T98G cells were transfected with V5-tagged FKBP9 wide type and mutants, and then treated with Tg. The ratios of V5 expression to their corresponding GAPDH were represented. **f** IB analysis for Caspase-12 and CHOP expression in SF-539-FKBP9-WT and SF-539-FKBP9-K265R cells exposed with Tg for 12 h. The ratios of Cleaved-Caspase-12, CHOP and V5 expression to their corresponding GAPDH were represented. All experiments in this figure were performed three times with comparable results

While studying the effect of Tg on GBM cells, we observed that endogenous FKBP9 protein levels in both SF-539 and T98G cells were downregulated upon Tg treatment as determined by IB (Additional file 5: Figure S5a). Tm treatment, to a lesser degree, also decreased FKBP9 protein levels in these cells (Additional file 5: Figure S5a). Of note, pretreatment with either GSK2606414 or ISRIB (both PERK inhibitors), but not 4μ8C (IRE1α inhibitor) could prevent Tg-induced downregulation of endogenous FKBP9 in SF-539 and T98G cells (Fig. 8a). These data suggested that FKBP9 levels were indeed downregulated upon ER stress. Further investigation revealed that the mRNA levels of FKBP9 were not significantly altered in SF-539 and T98G cells following Tg treatment (Additional file 6: Figure S6a). However, pretreatment with MG132 (a proteasome inhibitor) but not chloroquine (CQ) or bafilomycin A1 (BafA1) robustly antagonized the effect of Tg on the protein levels of FKBP9 in both SF-539 and T98G cells (Fig. 8b). In addition, FKBP9 ectopically expressed in GBM cells was also downregulated upon exposure to Tg (Fig. 8e). Together, these findings suggest that the proteasomal degradation pathway might be responsible for Tg-triggered downregulation of FKBP9 in GBM cells. Supporting this notion, Tg treatment induced pronounced ubiquitination of ectopic FKBP9 in transfected HEK293T cells or endogenous FKBP9 in SF-539 cells compared to the control (Fig. 8c). Moreover, among the three predicted lysine sites for FKBP9 ubiquitination [25], mutation of lysine to arginine at K265 but not K525 or K527 in FKBP9 robustly attenuated Tg-induced ubiquitination of FKBP9 (Fig. 8d). Accordingly, ectopic expression of the FKBP9-K265R mutant was more stable than its wild type counterpart upon Tg-induced stress (Fig. 8e). In addition, Tg-triggered activation of UPR signaling together with the expression of CHOP and cleavage of Caspase-12 were dramatically attenuated in SF-539 cells ectopically expressing the FKBP9-K265R mutant compared to cells expressing wt-FKBP9 (Fig. 8f). Consistent with these findings, ectopic expression of the FKBP9-K265R mutant rendered SF-539 cells less susceptible to Tg-induced cell death than wt-FKBP9 (Fig. 8f). Moreover, the FKBP9-K265R mutant exhibited a significantly increased capacity to promote cell growth in SF-539 cells compared to wt-FKBP9 (Additional file 6: Figure S6b).

## Discussion

In this study, we have investigated the roles and mechanism of action of FKBP9 in GBM in vitro and in vivo. We provide evidence that FKBP9 promotes the malignant phenotypes of GBM cells in vitro and in vivo. Mechanistically, p38MAPK signaling plays a critical role in FKBP9-mediated oncogenic activities. In addition, we demonstrate that as an ER-resident molecule, FKBP9 modulates the IRE1α-XBP1 pathway and confers GBM cell resistance to ER stress inducers that trigger FKBP9 ubiquitination and degradation. Moreover, high FKBP9 expression correlates with poor prognosis of GBM patients. Therefore, these findings suggest that FKBP9 plays an oncogenic role in GBM and is a novel regulator in UPR signaling.

Several members of the FKBP family are reported to reside in the ER and have been implicated in cancer [26–30]. Among the ER-resident FKBPs, the role of FKBP9 in cancer remains largely unknown. Our initial survey of the cBio Cancer Genomics Portal revealed that FKBP9 is amplified in gliomas, suggesting that FKBP9 might play a role in GBM biology. Bioinformatics analyses of available different public datasets all indicated that FKBP9 is upregulated in human high-grade gliomas and that high FKBP9 expression correlates with reduced overall survival of GBM patients, further supporting a role for FKBP9 in GBM. Moreover, both loss-of-function and gain-of-function studies demonstrated an oncogenic role of FKBP9 in GBM progression, which was further confirmed in both CAM and mouse xenograft models. Further mechanistic demonstrated that FKBP9 expression regulated the activation of p38MAPK via ASK1. Moreover, the ASK1-p38 signaling contributed to FKBP9 expression-mediated function in GBM cells. Of interest, an M541I variant of FKBP9 was found in clinical glioma tissue samples [13]. However, the effects of the M541I mutation on FKBP9 expression and function have not been described. The M541I variant of FKBP9 failed to activate p38MAPK as wild-type FKBP9 did. We found that the M541I mutation impaired FKBP9-mediated oncogenic activities in GBM cells. Further study is needed to determine the clinical relevance of this FKBP variant in patients with GBM.

One of the important findings of our study is that FKBP9 might be a critical mediator of UPR signaling in GBM cells. We found that depletion of FKBP9 activated the IRE1α-XBP1 pathway in GBM cells, a key branch of UPR signaling. The UPR is often triggered in response to ER stress caused by the accumulation of misfolded or unfolded proteins [31]. We found that depletion of FKBP9 enhanced aggresome accumulation in GBM cells, indicating that FKBP9 expression perturbs the accumulation of misfolded proteins, which leads to the deregulation of UPR signaling. Excessive activation of the UPR in cancer cells can lead to cell growth inhibition and even cell death [32]. Blocking the activation of the IRE1α-XBP1 pathway in FKBP9-depleted GBM cells with a small molecule inhibitor could impair the effects of FKBP9 depletion on GBM cell growth, supporting a role for the UPR signaling in FKBP9-mediated activities in GBM. The IRE1α pathway has been implicated in various models of experimental GBM [20, 33, 34] and contributes to GBM growth and vascularization [35, 36]. However, Lhomond et al. recently reported that IRE1α-XBP1 downstream signals play antagonistic roles in cancer progression, whereby XBP1s provides protumoral signals, whereas RNA regulated IRE1-dependent decay (RIDD) of mRNA and miR-17 elicits antitumoral effects [37]. Together, these studies highlight a complex role of the IRE1α-XBP1 pathway in GBM development.

Recent studies have suggested ER stress-inducing drugs alone or in combination with temozolomide (TMZ) as an alternative therapeutic strategy for GBM [38, 39]. In addition, Wang and Kaufman proposed a hypothesis that interfering with basal UPR signaling in cancer cells (either increase or decrease) will lead to an antitumor effect [31]. We found that FKBP9 expression conferred GBM cell resistance to ER stress inducers. Therefore, FKBP9 expression might be exploited as a predictor to evaluate the effects of ER stress inducers on GBM in future clinical settings. In addition, ER stress inducers caused FKBP9 degradation in GBM cells in a proteasomal-dependent manner. Of importance, inhibition of FKBP9 ubiquitination could attenuate the FKBP9-mediated oncogenic effects on GBM cells.

## Conclusions

Taken together, our data indicated the carcinogenesis of FKBP9 in glioma, and revealed that knockdown of FKBP9 causes ER stress. We further found that FKBP9 expression confers resistance to ER stress inducer-triggered cell death. Our results also showed that FKBP9 could be degraded by ubiquitination with Tg treatment, which would implicate a novel therapeutic avenue for the treatment of glioma.

## Supplementary information

**Additional file 1: Figure S1.** a Amplification and mutation analysis of FKBP9 across all types of cancer from http://www.cbioportal.org. b IB analysis for FKBP9, Sox2, Oct4 and Nanog protein levels in 2D and 3D cultured cells of two additional independent experiments. α-Tubulin was used as a loading control. c Correlation of FKBP9 mRNA levels with Sox2, Oct4 and Nestin from CGGA, TCGA or REMBRENDT. d T98G and U-87 MG cells were transfected with V5-tagged FKBP9 wide type or ER sequence-deleted mutation. IF assays for V5 (red), calnexin (green) and DAPI (blue) in the transfected T98G and U-87 MG cells. Representative merged images were also shown for fluorescence signals. Scale bar = 25 μm.

**Additional file 2: Figure S2.** a SF-539-shFKBP9 and T98G-shFKBP9 cells were introduced with adenoviruses-control (Ad-vector) and adenoviruses-expressing FKBP9 (Ad-FKBP9). Protein levels of Bcl-2, XIAP and Mcl-1 detected by IB were shown as two additional independent experiments. b The ratios of Bcl-2, XIAP and Mcl-1 expression to their corresponding GAPDH were represented. (**p < 0.01).

**Additional file 3: Figure S3.** a Analysis of key proteins expression of AKT-mTOR, Wnt, JAK-STAT, Hippo-YAP and NF-κB pathways in shControl, shFKBP9, and Ad-FKBP9 rescued groups of SF-539 and T98G cells by IB assay. b IB analysis of FKBP9-WT and M541I mutation overexpressing cells for key proteins of AKT-mTOR, Wnt, NF-κB, JAK-STAT and Hippo-YAP pathways. c Analysis of colony formation of SF-539-FKBP9-WT cells treated with vehicle or 2.5 μM NQDI-1. SF-539-FKBP9-WT cells were transfected with two siRNA duplexes targeting ASK1 (siASK1) or control siRNA (siCtrl) for 48 h, colony formation assays were performed. IB analysis for ASK1 and pHSP27 expression with GAPDH as a loading control.

**Additional file 4: Figure S4.** a IB analysis for pIRE1α in stable FKBP9-depleted SF-539 and T98G cells transfected with two siRNAs targeting p38.

**Additional file 5: Figure S5.** a IB analysis for BiP, CHOP and FKBP9 expression of SF-539 and T98G cells exposed to Tg or Tm for 6, 12, 24 h. GAPDH was used as a loading control. b Analysis of colony formation of SF539-FKBP9 and T98G-FKBP9 cells (Tm, 0.5 μM). IB analysis for Caspase-12, CHOP and BiP expression in SF539-FKBP9 and T98G-FKBP9 cells treated with Tm for 12 h. c Analysis of colony formation of SF539-FKBP9-WT/M541I and T98G-FKBP9-WT/M541I cells (Tg, 0.1 μM; Tm, 0.5 μM). IB analysis for CHOP, Bcl-xL and XIAP expression in these cells treated with Tg for 12 h. d Cell viability analysis of SF-539-shFKBP9 and T98G-shFKBP9 cells exposed to Tg (0.2 μM) or Tm (1.2 μM) for 24, 48 and 72 h by CCK8, respectively. Analysis of colony formation of SF539-shFKBP9 and T98G-shFKBP9 cells. (Tg, 0.1 μM; Tm, 0.5 μM).

**Additional file 6: Figure S6.** a RT-PCR analysis for FKBP9 mRNA levels of Tg-treated SF-539 and T98G cells. b Analysis of colony and spheroid forming abilities of SF-539-FKBP9-WT and SF-539-FKBP9-K265R cells. Data are represented as mean ± S.D. (**p < 0.01, ***p < 0.001)

**Additional file 7: Table S1.** Case information and pathological diagnosis of glioma patient tissue sections. Case information and pathological diagnosis were provided by the Second Affiliated Hospital of Dalian Medical University.

**Additional file 8: Table S2.** The list of significant transcripts that caused by FKBP9 depletion of SF-539 cells. (*p* value< 0.05, log2 fold change> 1). RNA was extracted from shControl and shFKBP9 cells and RNA-Seq was performed by the Novogene Corporation (Beijing, China). The list of significant transcripts that caused by FKBP9 depletion of SF-539 cells was shown.

**Additional file 9: Table S3.** The FPKM value list of upregulated transcripts related to ER stress. RNA was extracted from shControl and shFKBP9 cells and RNA-Seq was performed by the Novogene Corporation (Beijing, China).The FPKM value list of transcripts that caused by FKBP9 depletion of SF-539 cells was shown.

## Data Availability

All data during this study are included within this published article and additional files. Any material described in the article can be requested directly from corresponding author on reasonable request.

## Abbreviations

3D: Three dimensional
Baf A1: Bafalomycin A1
CAM: Chick embryo’s chorioallantoic membrane
CCGA: Chinese Glioma Genome Atlas
CQ: Chloroquine
ER stress: Endoplasmic reticulum stress
FKBP9: FK506-binding protein 9
GBM: Glioblastoma
HGG: High grade gliomas
IB: Immunoblotting
IHC: Immunohistochemistry
IP: Immunoprecipitation
IRE1α: Inositol requiring enzyme 1α
LGG: Low grade glioma
qRT-PCR: Quantitative real time PCR
TCGA: The Cancer Genome Atlas
Tg: Thapsigargin
Tm: Tunicamycin
TMZ: Temozolomide
UPR: Unfolded protein response
WT: Wild type
XBP1: X box-binding protein-1

## References

[1] Ghartey-Kwansah G, Li Z, Feng R, Wang L, Zhou X, Chen FZ, Xu MM, Jones O, Mu Y, Chen S (2018). Comparative analysis of FKBP family protein: evaluation, structure, and function in mammals and Drosophila melanogaster. BMC Dev Biol.

[2] Prakash A, Shin J, Rajan S, Yoon HS (2016). Structural basis of nucleic acid recognition by FK506-binding protein 25 (FKBP25), a nuclear immunophilin. Nucleic Acids Res.

[3] Jiang W, Cazacu S, Xiang C, Zenklusen JC, Fine HA, Berens M, Armstrong B, Brodie C, Mikkelsen T (2008). FK506 binding protein mediates glioma cell growth and sensitivity to rapamycin treatment by regulating NF-kappaB signaling pathway. Neoplasia.

[4] Romano S, Staibano S, Greco A, Brunetti A, Nappo G, Ilardi G, Martinelli R, Sorrentino A, Di Pace A, Mascolo M (2013). FK506 binding protein 51 positively regulates melanoma stemness and metastatic potential. Cell Death Dis.

[5] Romano S, Xiao Y, Nakaya M, D'Angelillo A, Chang M, Jin J, Hausch F, Masullo M, Feng X, Romano MF (2015). FKBP51 employs both scaffold and isomerase functions to promote NF-kappaB activation in melanoma. Nucleic Acids Res.

[6] Lagadari M, Zgajnar NR, Gallo LI, Galigniana MD (2016). Hsp90-binding immunophilin FKBP51 forms complexes with hTERT enhancing telomerase activity. Mol Oncol.

[7] Srivastava SK, Bhardwaj A, Arora S, Tyagi N, Singh AP, Carter JE, Scammell JG, Fodstad O, Singh S (2015). Interleukin-8 is a key mediator of FKBP51-induced melanoma growth, angiogenesis and metastasis. Br J Cancer.

[8] Liu T, Xiong J, Yi S, Zhang H, Zhou S, Gu L, Zhou M (2017). FKBP12 enhances sensitivity to chemotherapy-induced cancer cell apoptosis by inhibiting MDM2. Oncogene.

[9] Quinn MC, Wojnarowicz PM, Pickett A, Provencher DM, Mes-Masson AM, Davis EC, Tonin PN (2013). FKBP10/FKBP65 expression in high-grade ovarian serous carcinoma and its association with patient outcome. Int J Oncol.

[10] Hagedorn M, Siegfried G, Hooks KB, Khatib AM (2016). Integration of zebrafish fin regeneration genes with expression data of human tumors in silico uncovers potential novel melanoma markers. Oncotarget.

[11] Ge Y, Xu A, Zhang M, Xiong H, Fang L, Zhang X, Liu C, Wu S (2017). FK506 binding protein 10 is overexpressed and promotes renal cell carcinoma. Urol Int.

[12] Cerami E, Gao J, Dogrusoz U, Gross BE, Sumer SO, Aksoy BA, Jacobsen A, Byrne CJ, Heuer ML, Larsson E (2012). The cBio cancer genomics portal: an open platform for exploring multidimensional cancer genomics data. Cancer Discovery.

[13] Verhaak RG, Hoadley KA, Purdom E, Wang V, Qi Y, Wilkerson MD, Miller CR, Ding L, Golub T, Mesirov JP (2010). Integrated genomic analysis identifies clinically relevant subtypes of glioblastoma characterized by abnormalities in PDGFRA, IDH1, EGFR, and NF1. Cancer Cell.

[14] Suh YJ, Choe JY, Park HJ (2017). Malignancy in Pheochromocytoma or Paraganglioma: integrative analysis of 176 cases in TCGA. Endocr Pathol.

[15] Kim I, Xu W, Reed JC (2008). Cell death and endoplasmic reticulum stress: disease relevance and therapeutic opportunities. Nat Rev Drug Discov.

[16] Kang BR, Yang SH, Chung BR, Kim W, Kim Y (2016). Cell surface GRP78 as a biomarker and target for suppressing glioma cells. Sci Rep.

[17] Shuda M, Kondoh N, Imazeki N, Tanaka K, Okada T, Mori K, Hada A, Arai M, Wakatsuki T, Matsubara O (2003). Activation of the ATF6, XBP1 and grp78 genes in human hepatocellular carcinoma: a possible involvement of the ER stress pathway in hepatocarcinogenesis. J Hepatol.

[18] Zheng HC, Takahashi H, Li XH, Hara T, Masuda S, Guan YF, Takano Y (2008). Overexpression of GRP78 and GRP94 are markers for aggressive behavior and poor prognosis in gastric carcinomas. Hum Pathol.

[19] Tameire F, Verginadis KC (2015). Cell intrinsic and extrinsic activators of the unfolded protein response in cancer: mechanisms and targets for therapy. Semin Cancer Biol.

[20] Obacz Joanna, Avril Tony, Le Reste Pierre-Jean, Urra Hery, Quillien Véronique, Hetz Claudio, Chevet Eric (2017). Endoplasmic reticulum proteostasis in glioblastoma—From molecular mechanisms to therapeutic perspectives. Science Signaling.

[21] Ye T, Wei L, Shi J, Jiang K, Xu H, Hu L, Kong L, Zhang Y, Meng S, Piao H (2019). Sirtuin1 activator SRT2183 suppresses glioma cell growth involving activation of endoplasmic reticulum stress pathway. BMC Cancer.

[22] Meng S, Chen Z, Munoz-Antonia T, Wu J (2005). Participation of both Gab1 and Gab2 in the activation of the ERK/MAPK pathway by epidermal growth factor. Biochem J.

[23] Liu M, Jiang K, Lin G, Liu P, Yan Y, Ye T, Yao G, Barr MP, Liang D, Wang Y (2018). Ajuba inhibits hepatocellular carcinoma cell growth via targeting of beta-catenin and YAP signaling and is regulated by E3 ligase Hakai through neddylation. J Exp Clin Cancer Res.

[24] Kopito RR (2000). Aggresomes, inclusion bodies and protein aggregation. Trends Cell Biol.

[25] PhosphoSitePlus® [https://www.phosphosite.org. Accessed 20 Dec 2019].

[26] Ramadori G, Konstantinidou G, Venkateswaran N, Biscotti T, Morlock L, Galie M, Williams NS, Luchetti M, Santinelli A, Scaglioni PP (2015). Diet-induced unresolved ER stress hinders KRAS-driven lung tumorigenesis. Cell Metab.

[27] Huang Z, Li J, Du S, Tang Y, Huang L, Xiao L, Tong P (2016). FKBP14 overexpression contributes to osteosarcoma carcinogenesis and indicates poor survival outcome. Oncotarget.

[28] Kim KH, Yeo SG, Yoo BC, Myung JK (2017). Identification of calgranulin B interacting proteins and network analysis in gastrointestinal cancer cells. PLoS One.

[29] Garrido MF, Martin NJ, Bertrand M, Gaudin C, Commo F, El Kalaany N, Al Nakouzi N, Fazli L, Del Nery E, Camonis J (2019). Regulation of eIF4F translation initiation complex by the Peptidyl Prolyl Isomerase FKBP7 in Taxane-resistant prostate Cancer. Cli Cancer Res.

[30] Lin IY, Yen CH, Liao YJ, Lin SE, Ma HP, Chan YJ, Chen YM (2013). Identification of FKBP11 as a biomarker for hepatocellular carcinoma. Anticancer Res.

[31] Wang M, Kaufman RJ (2014). The impact of the endoplasmic reticulum protein-folding environment on cancer development. Nat Rev Cancer.

[32] Hsu Sheng-Kai, Chiu Chien-Chih, Dahms Hans-Uwe, Chou Chon-Kit, Cheng Chih-Mei, Chang Wen-Tsan, Cheng Kai-Chun, Wang Hui-Min David, Lin I-Ling (2019). Unfolded Protein Response (UPR) in Survival, Dormancy, Immunosuppression, Metastasis, and Treatments of Cancer Cells. International Journal of Molecular Sciences.

[33] Lhomond S, Pallares N, Barroso K, Schmit K, Dejeans N, Fazli H, Taouji S, Patterson JB, Chevet E, Oslowski CM (2015). Adaptation of the Secretory Pathway in Cancer Through IRE1 Signaling. Stress Responses: Methods and Protocols.

[34] Jabouille A, Delugin M, Pineau R, Dubrac A, Soulet F, Lhomond S, Pallares-Lupon N, Prats H, Bikfalvi A, Chevet E, et al. Glioblastoma invasion and cooption depend on IRE1alpha endoribonuclease activity. Oncotarget 2015;6(28):24922-34. 10.18632/oncotarget.4679 PMC4694804 26325176

[35] Dejeans N, Pluquet O, Lhomond S, Grise F, Bouchecareilh M, Juin A, Meynard-Cadars M, Bidaud-Meynard A, Gentil C, Moreau V (2012). Autocrine control of glioma cells adhesion and migration through IRE1alpha-mediated cleavage of SPARC mRNA. J Cell Sci.

[36] Pluquet O, Dejeans N, Bouchecareilh M, Lhomond S, Pineau R, Higa A, Delugin M, Combe C, Loriot S, Cubel G (2013). Posttranscriptional regulation of PER1 underlies the oncogenic function of IREalpha. Cancer Res.

[37] Lhomond S, Avril T, Dejeans N, Voutetakis K, Doultsinos D, McMahon M, Pineau R, Obacz J, Papadodima O, Jouan F, et al. Dual IRE1 RNase functions dictate glioblastoma development. EMBO Mol Med. 2018;10(3):e7929. 10.15252/emmm.201707929 PMC5840541 29311133

[38] Xipell E, Aragon T, Martinez-Velez N, Vera B, Idoate MA, Martinez-Irujo JJ, Garzon AG, Gonzalez-Huarriz M, Acanda AM, Jones C (2016). Endoplasmic reticulum stress-inducing drugs sensitize glioma cells to temozolomide through downregulation of MGMT, MPG, and Rad51. Neuro-oncology.

[39] Martinez NJ, Rai G, Yasgar A, Lea WA, Sun H, Wang Y, Luci DK, Yang SM, Nishihara K, Takeda S (2016). A High-Throughput Screen Identifies 2,9-Diazaspiro[5.5]Undecanes as Inducers of the Endoplasmic Reticulum Stress Response with Cytotoxic Activity in 3D Glioma Cell Models. PloS one.

