# Supplementary figures and images for: FKBP9 promotes the malignant behavior of glioblastoma cells and confers resistance to endoplasmic reticulum stress inducers
Source: J Exp Clin Cancer Res. 2020 Feb 28;39:44. doi: 10.1186/s13046-020-1541-0 (PMC7048151; doi:10.1186/s13046-020-1541-0)

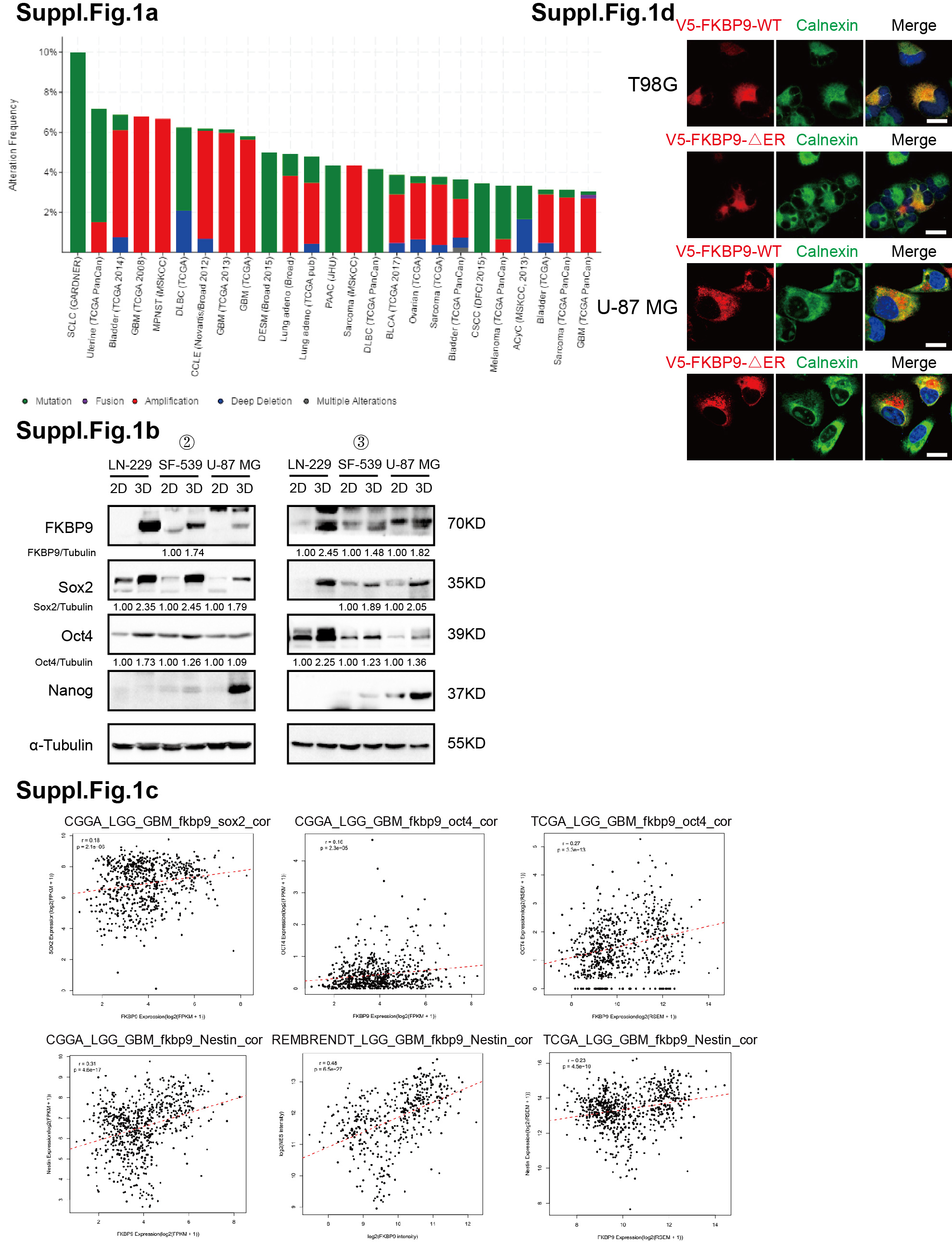

Supplement: Supplementary file 1 — Additional file 1: Figure S1. a Amplification and mutation analysis of FKBP9 across all types of cancer from http://www.cbioportal.org. b IB analysis for FKBP9, Sox2, Oct4 and Nanog protein levels in 2D and 3D cultured cells of two additional independent experiments. α-Tubulin was used as a loading control. c Correlation of FKBP9 mRNA levels with Sox2, Oct4 and Nestin from CGGA, TCGA or REMBRENDT. d T98G and U-87 MG cells were transfected with V5-tagged FKBP9 wide type or ER sequence-deleted mutation. IF assays for V5 (red), calnexin (green) and DAPI (blue) in the transfected T98G and U-87 MG cells. Representative merged images were also shown for fluorescence signals. Scale bar = 25 μm. [file 13046_2020_1541_MOESM1_ESM.jpg]

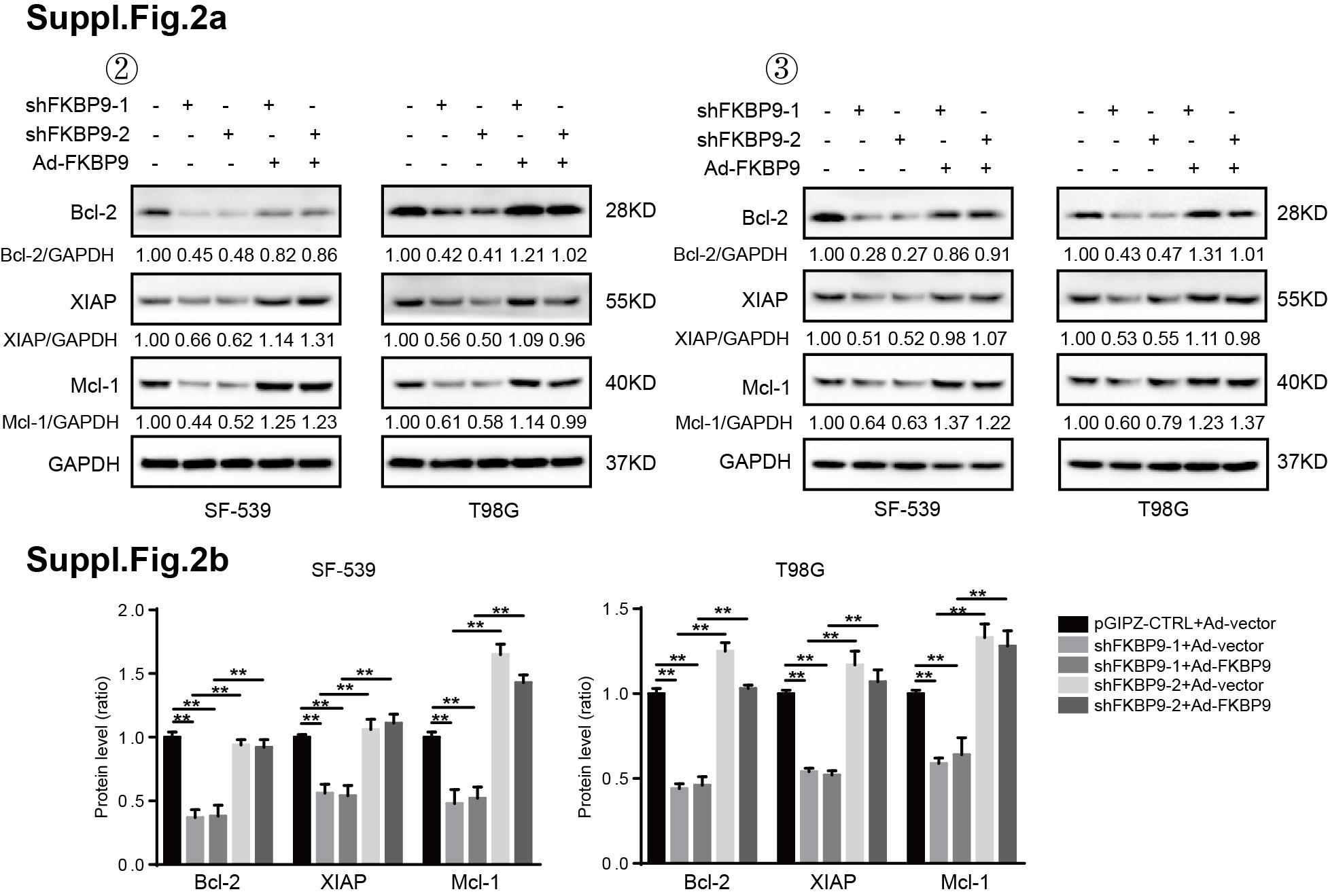

Supplement: Supplementary file 2 — Additional file 2: Figure S2. a SF-539-shFKBP9 and T98G-shFKBP9 cells were introduced with adenoviruses-control (Ad-vector) and adenoviruses-expressing FKBP9 (Ad-FKBP9). Protein levels of Bcl-2, XIAP and Mcl-1 detected by IB were shown as two additional independent experiments. b The ratios of Bcl-2, XIAP and Mcl-1 expression to their corresponding GAPDH were represented. (**p < 0.01). [file 13046_2020_1541_MOESM2_ESM.jpg]

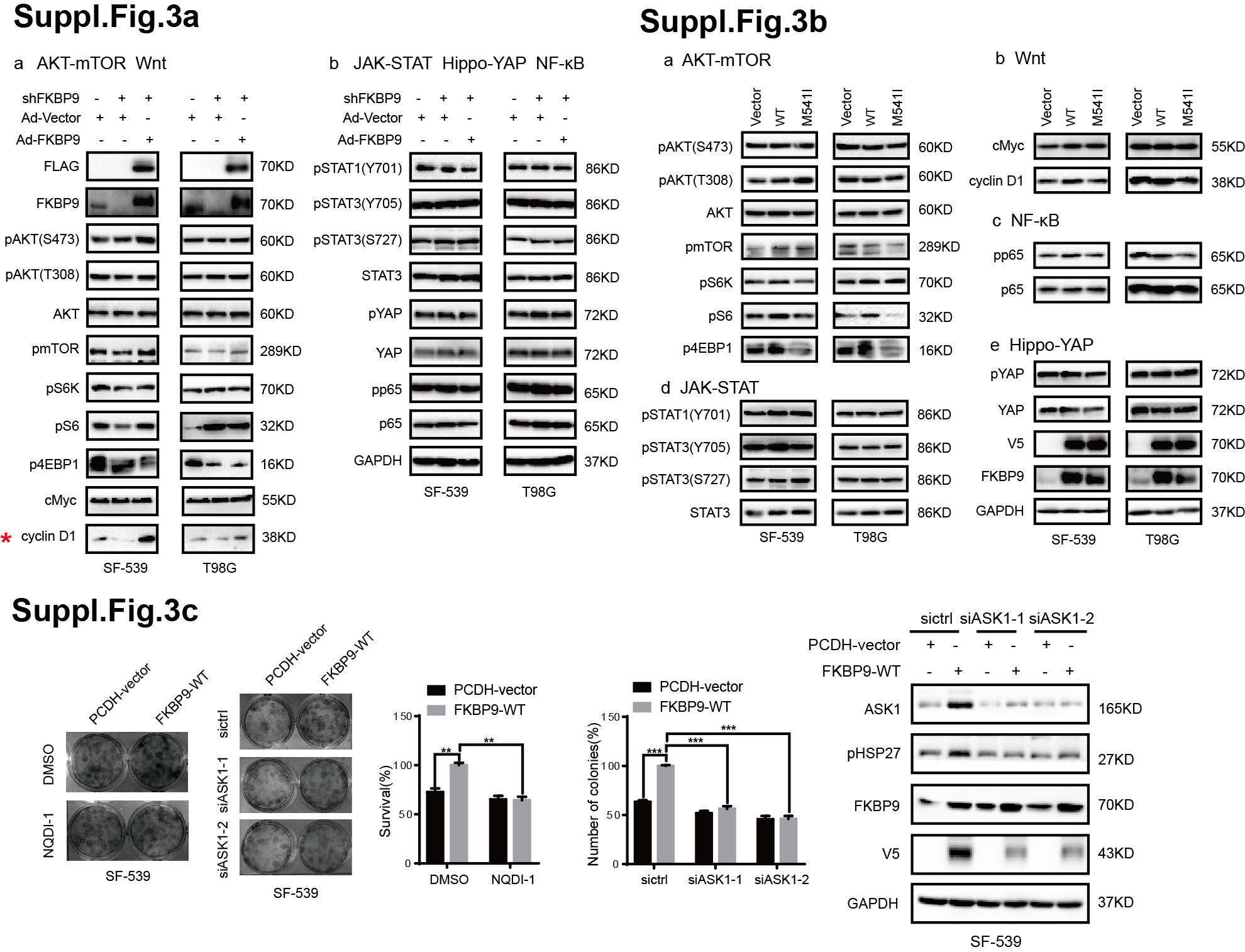

Supplement: Supplementary file 3 — Additional file 3: Figure S3. a Analysis of key proteins expression of AKT-mTOR, Wnt, JAK-STAT, Hippo-YAP and NF-κB pathways in shControl, shFKBP9, and Ad-FKBP9 rescued groups of SF-539 and T98G cells by IB assay. b IB analysis of FKBP9-WT and M541I mutation overexpressing cells for key proteins of AKT-mTOR, Wnt, NF-κB, JAK-STAT and Hippo-YAP pathways. c Analysis of colony formation of SF-539-FKBP9-WT cells treated with vehicle or 2.5 μM NQDI-1. SF-539-FKBP9-WT cells were transfected with two siRNA duplexes targeting ASK1 (siASK1) or control siRNA (siCtrl) for 48 h, colony formation assays were performed. IB analysis for ASK1 and pHSP27 expression with GAPDH as a loading control. [file 13046_2020_1541_MOESM3_ESM.jpg]

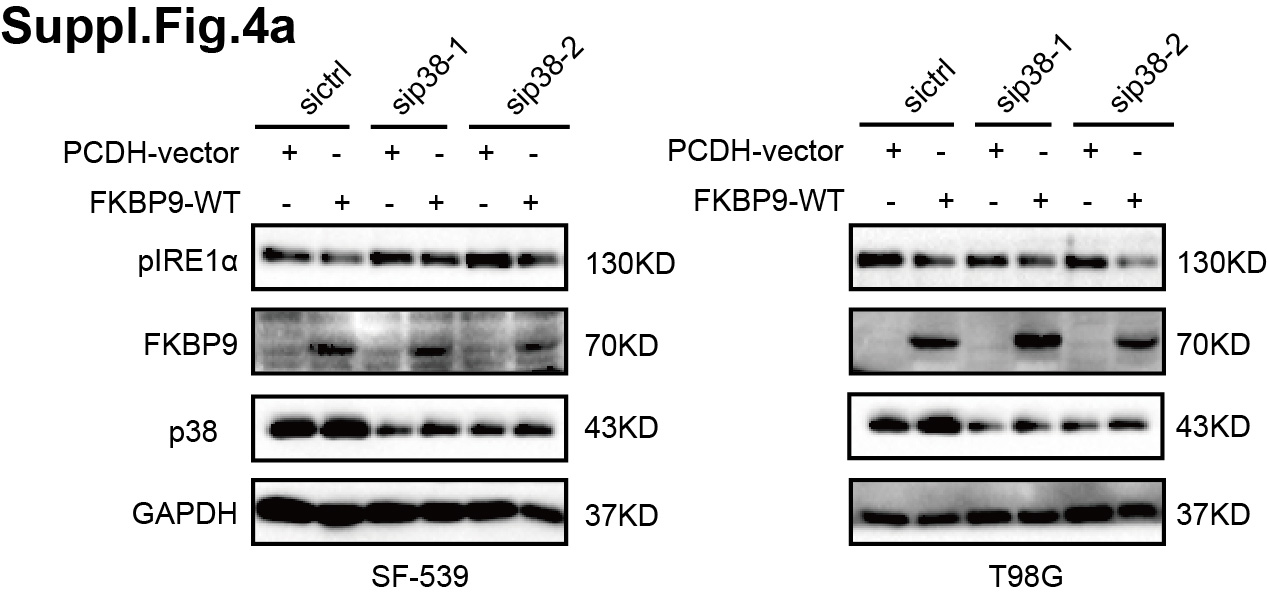

Supplement: Supplementary file 4 — Additional file 4: Figure S4. a IB analysis for pIRE1α in stable FKBP9-depleted SF-539 and T98G cells transfected with two siRNAs targeting p38. [file 13046_2020_1541_MOESM4_ESM.jpg]

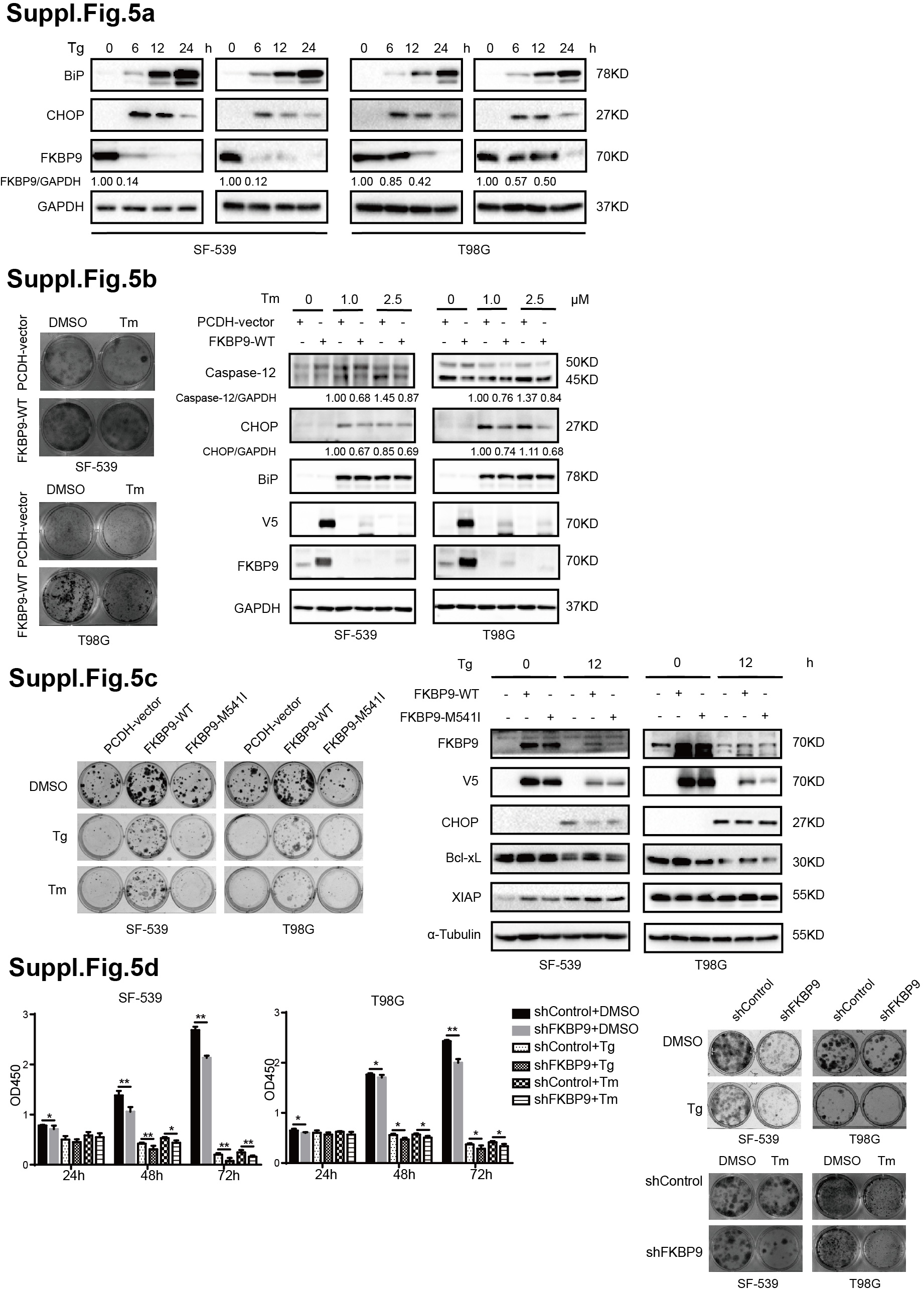

Supplement: Supplementary file 5 — Additional file 5: Figure S5. a IB analysis for BiP, CHOP and FKBP9 expression of SF-539 and T98G cells exposed to Tg or Tm for 6, 12, 24 h. GAPDH was used as a loading control. b Analysis of colony formation of SF539-FKBP9 and T98G-FKBP9 cells (Tm, 0.5 μM). IB analysis for Caspase-12, CHOP and BiP expression in SF539-FKBP9 and T98G-FKBP9 cells treated with Tm for 12 h. c Analysis of colony formation of SF539-FKBP9-WT/M541I and T98G-FKBP9-WT/M541I cells (Tg, 0.1 μM; Tm, 0.5 μM). IB analysis for CHOP, Bcl-xL and XIAP expression in these cells treated with Tg for 12 h. d Cell viability analysis of SF-539-shFKBP9 and T98G-shFKBP9 cells exposed to Tg (0.2 μM) or Tm (1.2 μM) for 24, 48 and 72 h by CCK8, respectively. Analysis of colony formation of SF539-shFKBP9 and T98G-shFKBP9 cells. (Tg, 0.1 μM; Tm, 0.5 μM). [file 13046_2020_1541_MOESM5_ESM.jpg]

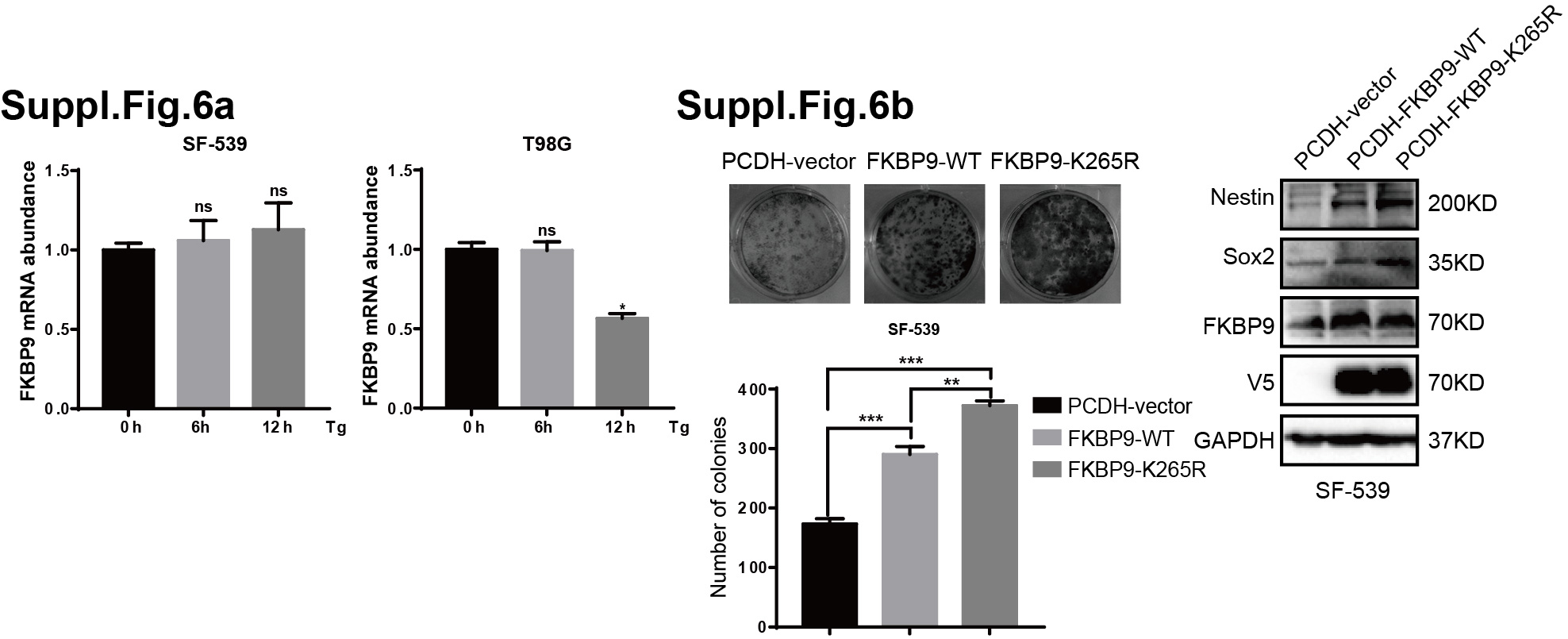

Supplement: Supplementary file 6 — Additional file 6: Figure S6. a RT-PCR analysis for FKBP9 mRNA levels of Tg-treated SF-539 and T98G cells. b Analysis of colony and spheroid forming abilities of SF-539-FKBP9-WT and SF-539-FKBP9-K265R cells. Data are represented as mean ± S.D. (**p < 0.01, ***p < 0.001) [file 13046_2020_1541_MOESM6_ESM.jpg]
